# Dung Beetles along a Tropical Altitudinal Gradient: Environmental Filtering on Taxonomic and Functional Diversity

**DOI:** 10.1371/journal.pone.0157442

**Published:** 2016-06-23

**Authors:** Cássio Alencar Nunes, Rodrigo Fagundes Braga, José Eugênio Cortes Figueira, Frederico de Siqueira Neves, G. Wilson Fernandes

**Affiliations:** 1 Laboratório de Ecologia de Populações, Departamento de Biologia Geral, Instituto de Ciências Biológicas, Universidade Federal de Minas Gerais, CP 486, 31270–901, Belo Horizonte, Minas Gerais, Brazil; 2 Laboratório de Ecologia e Conservação de Invertebrados, Departamento de Biologia, Setor de Ecologia, Universidade Federal de Lavras, CP 3037, 37200–000, Lavras, Minas Gerais, Brazil; 3 Laboratório de Ecologia de Insetos, Departamento de Biologia Geral, Instituto de Ciências Biológicas, Universidade Federal de Minas Gerais, CP 486, 31270–901, Belo Horizonte Minas Gerais, Brazil; 4 Ecologia Evolutiva & Biodiversidade /DBG, ICB/ Universidade Federal de Minas Gerais, CP 486, 31270–901, Belo Horizonte Minas Gerais, Brazil; Ben-Gurion University of the Negev, ISRAEL

## Abstract

Mountains provide an interesting context in which to study the many facets of biodiversity in response to macroclimate, since environmental conditions change rapidly due to elevation. Although the decrease in biodiversity with increasing elevation is generally accepted, our understanding of the variation of functional diversity along altitudinal gradients is still poorly known. The partitioning of diversity into spatial components can help to understand the processes that influence the distribution of species, and these studies are urgently needed in face of the increasing threats to mountain environments throughout the world. We describe the distribution of dung beetle diversity along an altitudinal gradient on a tropical mountain in southeastern Brazil, including the spatial partitioning of taxonomic and functional diversities. The altitudinal gradient ranged from 800 up to 1400 m a.s.l. and we collected dung beetles at every 100 m of altitude. We used the Rao Index to calculate γ, α and β diversity for taxonomic and functional diversity of dung beetles. Climatic, soil and vegetation variables were used to explain variation in community attributes along the altitudinal gradient. Dung beetle richness declined with altitude and was related to climatic and vegetation variables, but functional diversity did not follow the same pattern. Over 50% of γ taxonomic diversity was caused by among altitudes diversity (β), while almost 100% of functional diversity was due to the α component. Contrasting β taxonomic with β functional diversity, we suggest that there is ecological redundancy among communities and that the environment is filtering species in terms of the Grinnellian niche, rather than the Eltonian niche. β taxonomic diversity is caused mainly by the turnover component, reinforcing the hypothesis of environmental filtering. Global warming may have strong effects on mountain communities due to upslope range shifts and extinctions, and these events will lead to an even larger than previously expected loss of diversity as dung beetles γ taxonomic diversity is caused mainly by the β component.

## Introduction

Mountains provide an interesting context in which to learn how living beings respond to different macroclimates since they offer steep environmental gradients. Environmental conditions change rapidly with elevation on mountains [1,2], providing the opportunity to undertake ecological and evolutionary studies over short scales (e.g., [3]). Decades of research by ecologists and biogeographers have lead to a general hypothesis regarding species distribution along altitudinal gradients: diversity decreases with increasing elevation (e.g., [4–7]). In humid tropical mountains, species richness usually decreases monotonically with increasing altitude (e.g., [8,9]). The general geophysical and climatic trends with increasing altitude are: (i) decline of land area; (ii) decreasing total atmospheric pressure as well as partial pressure of O_2_ and CO_2_; (iii) reduction of air temperature; and (iv) increase in solar radiation [3]. Other factors can be associated with an altitudinal gradient, such as relative humidity, precipitation, wind velocity, geological substrates, nitrogen deposition and soil pH but they are driven by regional forces [3,10]. These are some of the mechanisms that influence the distribution of species of plants and animals on different mountains. As the abundance, diversity and functional traits of plants change along an altitudinal gradient, the primary production is affected, which can also affect animal distribution [10].

Biodiversity is a concept that includes not only species diversity (taxonomic) but also functional and phylogenetic diversity [11]. Functional diversity is one of the most important components of biodiversity that affects ecosystem functioning, and determining it can aid in the conservation of nature [12,13]. Studying both taxonomic diversity (TD) and functional diversity (FD) can improve the understanding of patterns of biodiversity since they capture different aspects of species ecological roles, resource use and habitat requirements. This, then, can lead to a better understanding of how environmental and biotic factors act as filters of species and their traits along a gradient [14,15]. Although there have been many studies focusing on patterns of species richness along altitudinal gradients, there is a lack of information regarding the other components of diversity along such gradients. A few number of studies showed that elevation can act as an important filter to diversity, however differently to TD and FD [14,16,17].

Beyond this multi-faceted concept, diversity can also be partitioned into different spatial components, which is crucial to understanding the processes that influence species distributions [18]. Regional diversity (called γ-diversity) can be partitioned into two components: diversity within local communities (α-diversity) and diversity among communities (β-diversity) [19,20]. Recently the partitioning of diversity has been extended to the other facets of diversity, such as FD [15,21–23], which has proven to be very useful in comparing taxonomic and functional facets of diversity at different spatial scales [24,25]. Additionally, β diversity (both TD and FD) can be partitioned into two main components: turnover (species replacement between communities) and nestedness (species loss or gain between communities) [26]. Studies that merge the multifaceted concept of diversity with spatial partitioning along environmental gradients are very rare (but see [12,25]), especially in tropical ecosystems, and as far as we know there is only one study involving altitudinal gradients [27]. Furthermore, the decomposition of both β-TD and β-FD is very rare in the literature (but see [28]).

Dung beetles are members of the subfamily Scarabaeinae (Coleoptera: Scarabaeidae), a diverse and abundant group of insects. They have been used widely as bioindicators of environmental conditions due to their sensibility to environmental changes and because they perform various ecological functions such as soil fertilization and aeration, increasing nutrient cycling and secondary seeds dispersal [29–35]. Several studies in Europe, North America, Southeast Asia, South Africa and in South America have revealed a consistent decrease in the number of species of dung beetles with increasing altitude (e.g., [36–40]), thereby corroborating the global trend in species distribution along altitudinal gradients. However, no study to date has examined the functional diversity of dung beetles along altitudinal gradients.

In this study we describe the dung beetle communities along a tropical altitudinal gradient in southeastern Brazil, including the spatial partitioning of taxonomic diversity (TD) and functional diversity (FD). We tested the hypothesis that environmental factors control the differentiation of diversity among and within communities. We focus on four scales: alpha diversity at the very local transect scale, beta diversity between transects in the same elevation belt, beta diversity between elevation belts and gamma diversity for the entire mountain. We expected a decrease in species richness with increasing altitude (e.g., [36]) and, as a consequence, a decrease in FD. We also expected that β-TD and β-FD of each elevation belt (within-elevation β) would diminish along the altitudinal gradient, because total diversity and habitat heterogeneity decreases with elevation. Furthermore, since an altitudinal gradient can provide different environmental filters (e.g., [3]), for both TD and FD, we expected β diversity-between elevations- to contribute more to γ diversity than α diversity. By partitioning β diversity we tested if mountaintop communities are nested sub-sets of lowland communities.

## Materials and Methods

### Study site

**Fig 1:**
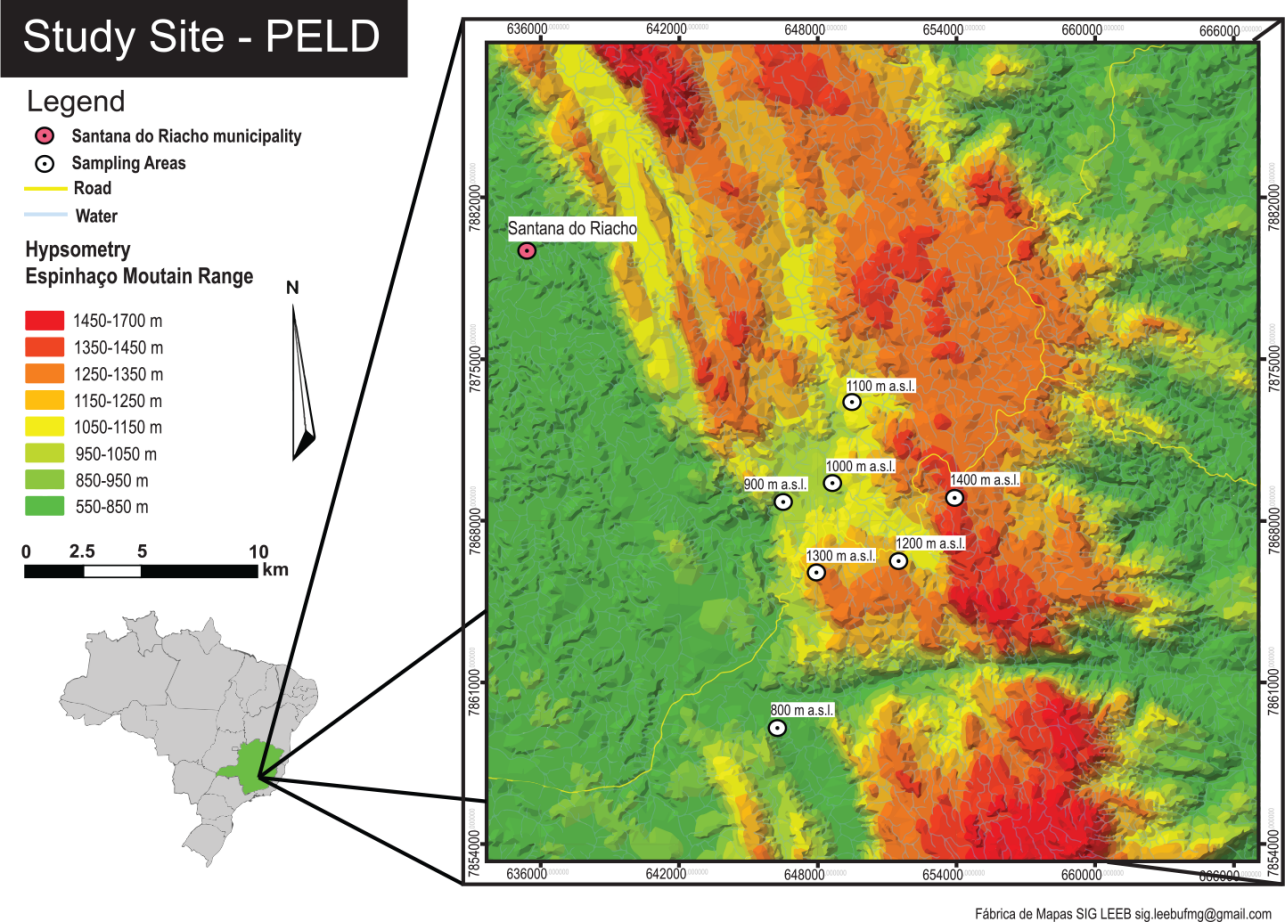
Altitudinal map of the study site with the seven sampling areas. Serra do Cipó, State of Minas Gerais, Brazil (Source: SIG–LEEB).

The study was conducted along an altitudinal gradient of cerrado and rupestrian grassland areas located in the southern part of the Espinhaço mountain range, in State of Minas Gerais, Brazil (19°10’ and 19°22’ S, 43°29’ and 43°36’ W) between September 2013 and June 2014 (Fig 1). The region, called Serra do Cipó, has a highland tropical Cwb Köppen climate with a rainy season between November and February and mean annual temperature and rainfall of 20°C and 1,500 mm, respectively [41]. The Espinhaço range is a quartzite mountain chain that crosses the southeast and part of northeast of Brazil and separates the Atlantic Forest and Cerrado biomes [42,43]. At the study location, soil and vegetation are very heterogeneous, varying among five principal habitats: peat bogs, sandy grasslands, rocky grasslands (rupestrian fields), rocky outcrops and cerrado [44]. Serra do Cipó is well known for its high plant and animal biodiversity and a large number of endemic species (e.g., [42,45–47]).

### Sampling Design and Environmental Variables

**Fig 2:**
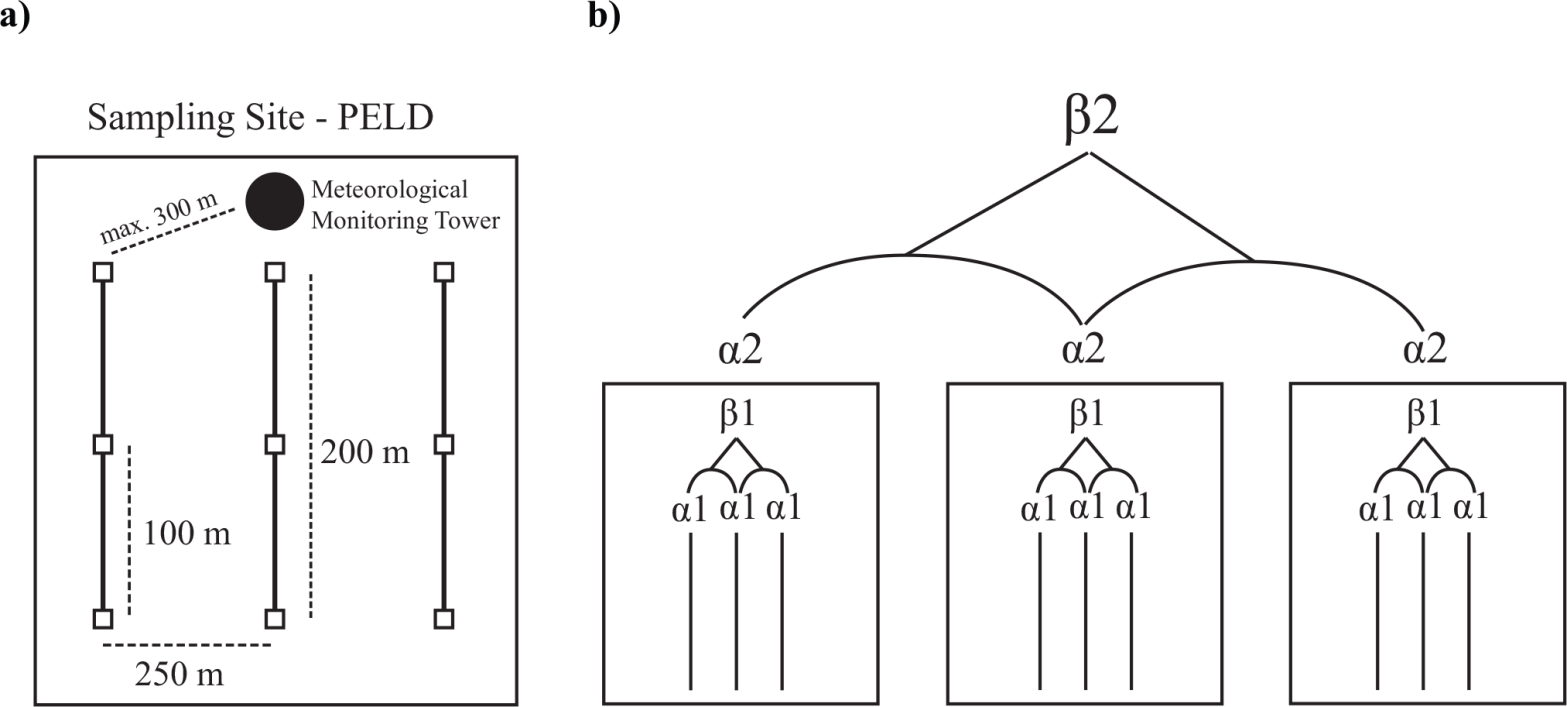
a) Schematic drawing of the sampling units that were replicated every 100 m of altitude along the altitudinal gradient at Serra do Cipó, State of Minas Gerais, Brazil (PELD–Long Term Ecological Research). Each sampling unit was composed by three 200 m long transects (solid lines) separated by 250 m. Within each transect we placed three traps (pitfalls) separated by 100 m (squares). The meteorological monitoring tower (black circle) was at the maximum distance of 300 m from transects; b) Diversity within transects (solid lines) is α1 diversity, diversity among transects is β1 diversity, diversity within an altitude (boxes) is α2 diversity and diversity among altitudes is β2 diversity.

This study is a part of a larger research project (Long Term Ecological Research–PELD–Site 17/Serra do Cipó) and we used its pre-established sampling areas to sample dung beetles and test our hypotheses. The altitudinal gradient ranged from 800 to 1400 m a.s.l., and sampling sites were distributed every 100 m of altitude (n = seven altitudes) with a minimum geographic distance of 2 km (see Fig 1). In each of these altitudinal sites we used three transects separated by at least 250 m, each consisting of three traps separated by 100 m (nine traps per altitude, 63 total, Fig 2A). We considered the altitude as our sampling unit.

At each altitude, a meteorological monitoring tower (equipped with the Onset HOBO^®^ U30 data-logger) recorded the following climatic parameters for the study period (between September 2013 and June 2014): air temperature, air humidity, soil humidity, solar radiation, and precipitation. The data-logger recorded the values every five minutes and we could obtain the mean, maximum, minimum and variation of the parameters. Furthermore, because the large project has multiple research lines, we were also able to acquire data on granulometry of the soil (see [48]) and vegetation structure; the richness, abundance, height and basal area of the plants (see [49]).

### Dung Beetle Community Attributes

Dung beetles were sampled during four time periods of the year: end of dry season (September 2013), beginning of rainy season (December 2013), end of rainy season (March 2014) and beginning of dry season (June 2014). To quantify dung beetle species richness, abundance and biomass we used baited pitfall traps. Traps were 9 cm deep and 15 cm in diameter, contained 250 ml of a salt + detergent solution, and were baited with 25 g of fresh human feces. Each trap was left in the field for 48 h, after which the beetles were collected, preserved and transported to the laboratory where all individuals were counted and identified to the lowest taxonomical level possible. We used an identification key to genera and subgenera [50] and its included taxonomic literature cited on the identification of New World species of Scarabaeinae. Species were assigned to a functional guild based on their food allocation strategies for reproduction [51]: rollers (telecoprids)—that construct balls where they will deposit their eggs and roll it away from the original food source (dung); tunnelers (paracoprids)—that dig tunnels directly beneath the food source where they will store their dung balls; and dwellers (endocoprids)—that live and reproduce inside the food source. To obtain the beetle biomass, all individuals were dried at 45°C to a constant weight and individually weighted in a 0.001 g precision scale. Using these weights we calculated the mean biomass of each species. Voucher specimens are deposited in the Evolutionary Ecology and Biodiversity Laboratory at Federal University of Minas Gerais. All necessary sampling permits were obtained for the described field studies. Responsible for the authorization: Sistema de Autorização e Informação em Biodiversidade (SISBIO); license number 38952–1, date 02/05/2013, authentication code: 47946752; http://www.icmbio.gov.br/sisbio/verificar-autenticidade.

### Data Analysis

#### Taxonomic and Functional Diversity

Dung beetle taxonomic diversity (TD) was measured by species richness and by the Simpson Index (incorporating abundance). To obtain functional diversity (FD) we calculated a species dissimilarity matrix based on multiple traits using the “Gower approach” from the “*trova*” function (see [52] for details) for R software [53]. We used mean biomass and functional guild for each dung beetle species for constructing the matrix, because these traits are considered to have the most effect on dung beetle ecological functions [31,54]. Gower distance is quite useful for combining different types of traits like quantitative (biomass of dung beetles) and qualitative (functional guild of dung beetles) [52]. We then used the dissimilarity matrix to calculate the Rao Index, which estimates FD based on species dissimilarities and abundances at each sampling point. We used the “Rao” R function [24] that calculates both the Simpson and Rao indexes taking into account Jost’s correction [55] with equivalent numbers for partition of diversity. With this function, it is possible to partition both TD and FD into α, β and γ diversity components, thus, providing “a standardized methodology applicable to compare the partition of different facets of diversity” [24].

α diversity is calculated by weighting each pair of species functional distances by their relative abundances:

$$\alpha_{Rao}=\sum d_{ij}p_{i}p_{j}\alpha$$

where *d*_*ij*_ is functional distance between two species, and *p*_*i*_ and *p*_*j*_ are the species relative abundance. If we consider *d*_*ij*_ = 1, which means that all species are different, the Rao Index becomes equivalent to the Simpson Index. γ diversity is calculated by the same formula, but pooling all local samples of a region. β diversity is the mean difference between regional and local communities. Applying Jost’s correction with equivalent numbers, we get β diversity independent of α. Using additive partitioning with equivalent numbers the formulas are:

$$\alpha_{corrected}=1/\left(1-\alpha_{Rao}\right);\;\gamma_{corrected}=1/\left(1-\gamma_{Rao}\right);\beta_{corrected}=\gamma_{corrected}-\alpha_{corrected}\beta$$

In this case α-TD is the number of equivalent species in a local community (minimum value = 1). Note that if all species have the same relative abundance in a sampling unit the Jost-corrected Simpson diversity equals the number of species. In the same way α-FD is the number of equivalent species (in terms of abundance) sharing no functional traits (minimum value = 1). The Jost-corrected Rao Index is maximal when all species in a sampling unit are maximally dissimilar and have equal abundances. β diversity is the average difference between local and regional diversity. To make β-TD and β-FD comparable we expressed β as a percentage of γ diversity (Proportional β = β_corrected_/γ_corrected_β; see [24] for more details on partitioning TD and FD).

To test our hypotheses, we partitioned diversity into α1 (diversity within transects), β1 (diversity among transects of the same altitude), α2 (diversity within an altitude), β2 (diversity among altitudes) and γ diversity (diversity of the entire altitudinal gradient) (Fig 2B). To obtain α diversity values we have summed all species that occur in each transect (α1) or altitude (α2).

To partition taxonomic and functional β diversity we used the method of multiple-sites similarity [26]. To do this, we used the “beta.multi” function of the “betapart” R package [53,56] (index used: Sørensen). We obtained turnover and nestedness components of β1 and β2diversity and represent them as a proportion. To obtain the β1 components we used transects of each altitude as units and to obtain the β2 components we used the data from the elevation belts (sampling sites). Note that this was a separate, but complimentary analysis that we used to obtain the components of β diversities and it was not mathematically related with the partition of γ diversity. However, conceptually, when we say that we did the partition of β1 and β2 diversities we are talking about the turnover and nestedness components of diversities among transects (β1) and among altitudes (β2).

#### Statistical Analysis

We summarized the various environmental variables using principal component analysis (PCA) on PAST 2.17 [57] and selected two axes for the climate and vegetation variables and two axes for soil (see details S1 Appendix). To analyze the effects of altitude and the environmental variables (summarized) on dung beetle abundance, richness and α-TD/FD, we used generalized linear models (GLMs) on R software [53]. Because the spatial patterns of diversity does not change along the year at Serra do Cipó (S4 Appendix), the data from all samples (four periods) of each area were pooled (summed), resulting in one value per altitude (α2, n = 7). We calculated the mean altitude of each area using the altitude of each sampling point. As we did with α2 component, β1-TD/FD and their components were analyzed with altitude and environmental variables. The residuals of all GLMs were analyzed to evaluate the adequacy of the error distribution and the minimally significant model was selected (some variables were *Quasipoisson* and some were *Gaussian*; details are presented in S3 Appendix).

We made a supplementary analysis of dung beetle communities along the altitudinal gradient using permutational multivariate analysis of variance (PERMANOVA). To do this, we used the Jaccard index as a dissimilarity measure and performed 999 permutations using the “adonis” function in the R package “vegan 2.0–7” [58]. We used the transect data to do the PERMANOVA. As in the other analysis, we constructed models with altitude and environmental variables as explanatory variables for the variation in dung beetle community composition.

We have constructed two null models to test if the observed values of α, β and γ values of TD and FD were different from what was expected by chance. To do this, we used the “permtafull” and “permatswap” functions of “vegan” R package [53,58]. In the first null model, we have used a presence-absence matrix of dung beetles species per transect, maintaining the number of species per transect and the number of transects that each species could occur (marginal sums of rows and columns). With this null model we could evaluate if the different spatial levels of TD and FD were different from expected by chance without considering abundance of species. In the other null model, we have used a count matrix with the abundance of dung beetles species per transect, maintaining the number of species per transect, the number of transect that each species could occur and the number of individuals per transect (support capacity of each local). With this second null model we could evaluate the role of evenness to TD and FD at different spatial levels.

## Results

We collected 3681 individuals of dung beetles representing 56 species. Paracoprids represented 75% of all the sampled species (42 species), telecoprids 16% (9 species) and endocoprids 9% (5 species) (details in S2 Appendix). At all altitudesthe first and second most abundant species together were at least three times more abundant than the third (at 1400 m a.s.l. they were 25 times!). In three of the seven altitude classes, most abundant species were at least twice more abundant than second. The most abundant functional group was at least twice more abundant than the next most abundant functional group in five of the altitude classes (at 1200 m a.s.l. it was 6 times).Small paracoprids were the dominant functional group at all altitudes, except for the lowest (800 m a.s.l.), where large paracoprids dominated (the details on the abundance ranking of species are also presented in S2 Appendix).

**Fig 3:**
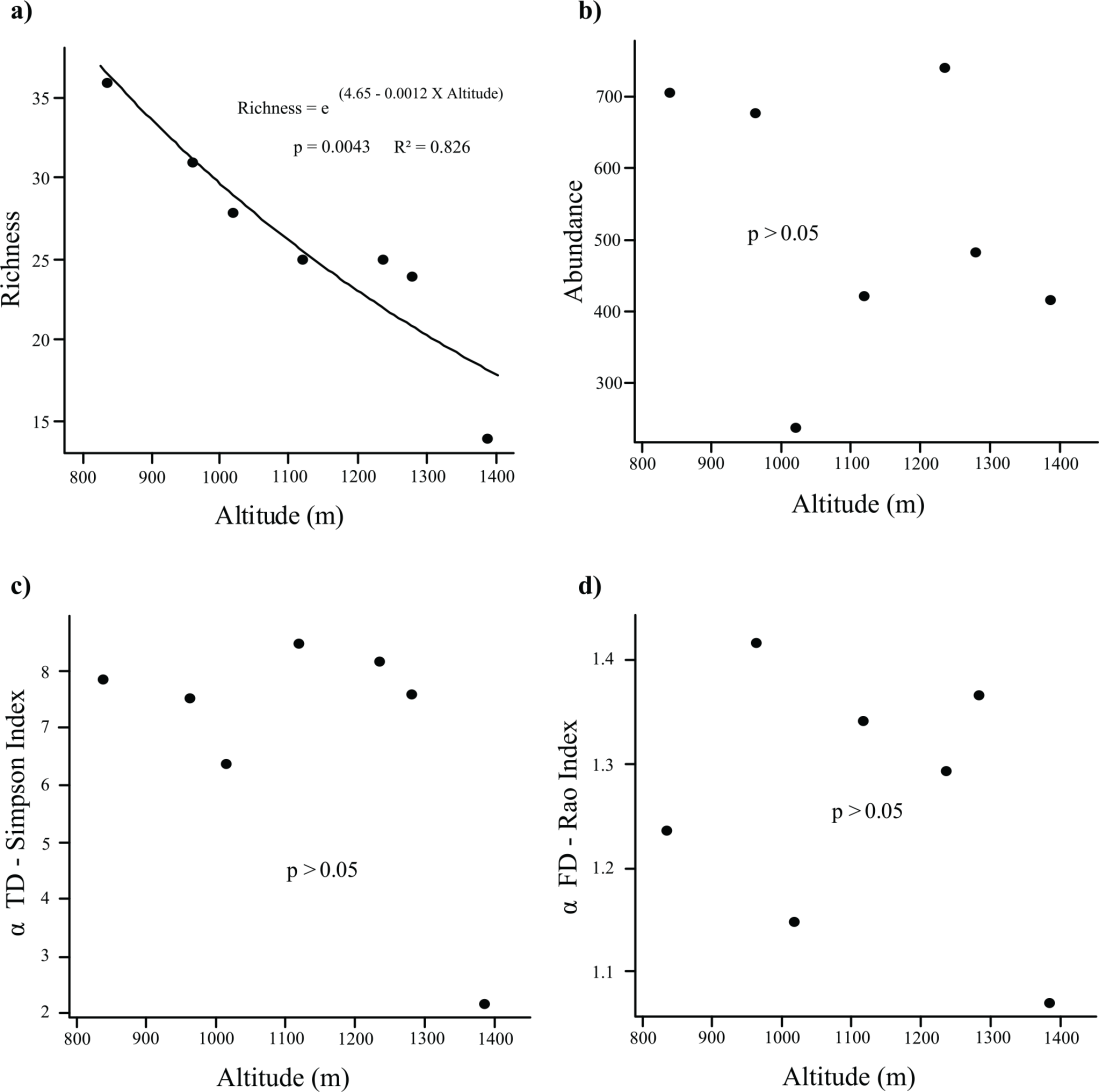
Dung beetles a) richness, b) abundance, c) α Simpson TD and d) α Rao FD along an altitudinal gradient at Serra do Cipó, State of Minas Gerais, Brazil.Each point denotes the diversity of the altitude (sampling unit).

Dung beetle richness declined with increasing in altitude (F = 24.311, p = 0.0043, R² = 0.82; Fig 3A). On the other hand, the altitudinal gradient did not influence beetle abundance (F = 0.365, p = 0.572; Fig 3B), even when we used the environmental factors as explanatory variables (F = 0.318, p = 0.848). Reduction of species richness was mainly driven by climatic variables such as temperature, radiation and humidity, followed by vegetation variables (Climatic-vegetation axis 1 in a minimal model) (F = 61.099, p = 0.0005). Climatic-vegetation axis 1 can be interpreted as a thermal-humidity-vegetation axis that is negatively correlated with altitude (S1 Appendix). Dung beetle richness declines with decreasing temperatures and increasing humidity and was higher when plant richness and abundance were higher.

Despite the fact that richness declined with increasing altitude, α Simpson TD was not influenced by the altitude (F = 1.8473, p = 0.2322; Fig 3C), nor by the environmental variables (F = 7.2026, p = 0.1256). Likewise, neither altitude (F = 0.328, p = 0.5916) nor the environmental variables (F = 0.516, p = 0.7421) influenced α Rao FD (Fig 3D). The detailed results of all GLMs performed in this study are summarized in S3 Appendix.

**Fig 4:**
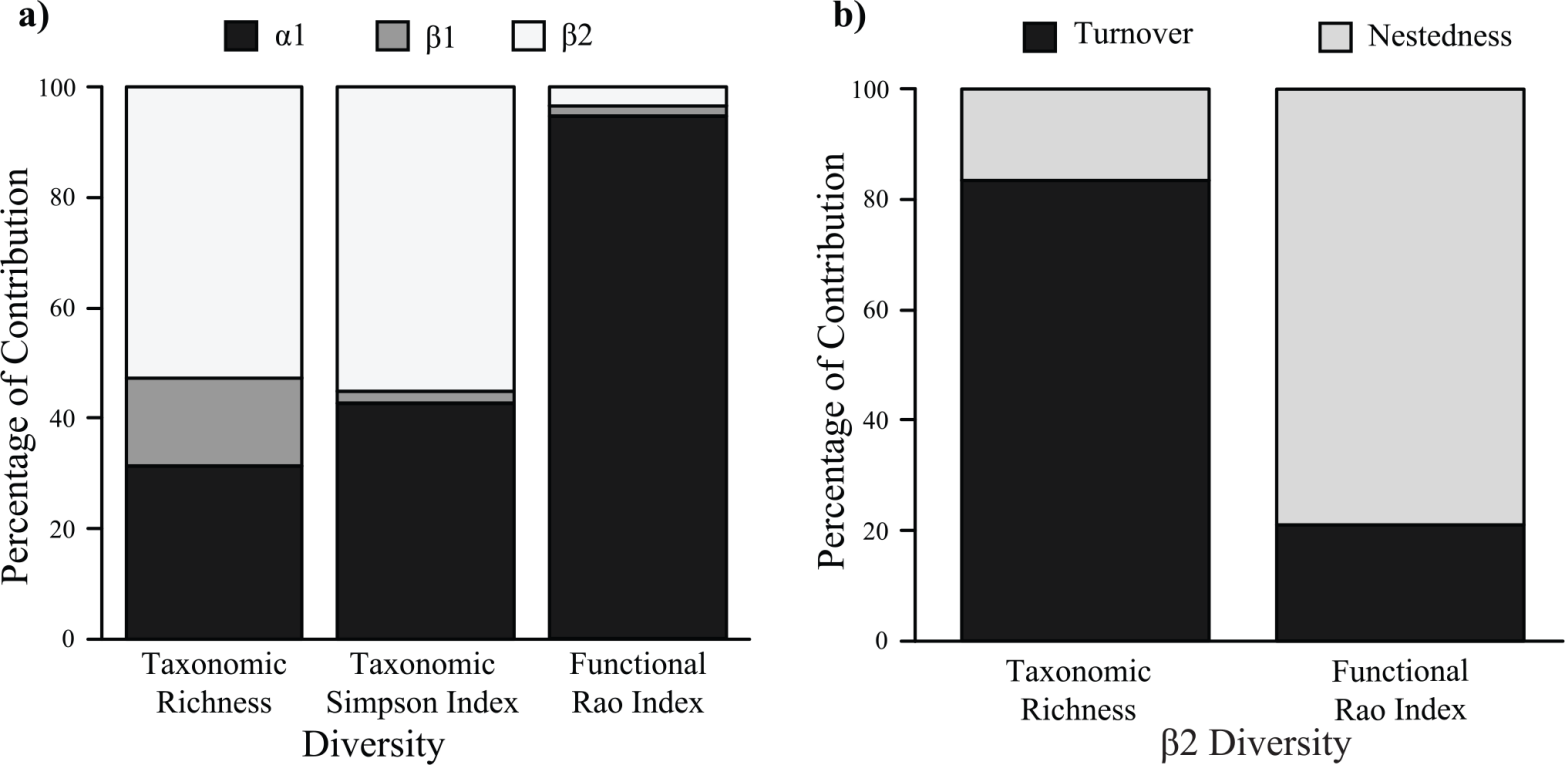
a) Contribution of α1, β1 and β2 to γ taxonomic and functional diversities. α1 is transects diversity, β1 is diversity among transects and β2 is diversity among altitudes. α2 (diversity of the altitude) is obtained by adding α1 to β1; b) Contribution of turnover and nestedness to β2 taxonomic and functional diversity; values obtained with the complimentary analysis of multiple-site similarity.

Fig 4A shows the contribution of α and β components to the taxonomic and functional γ diversity and Fig 4B shows the contribution of nestedness and turnover to the β2-TD/FD of the entire altitudinal gradient. The γ FD is caused almost completely by α1 component (94.7%), indicating that there is little difference in the FDs among the communities of different altitudes. On the other hand, 55% of γ TD is caused by the β2 component, which means that there are different community compositions along the altitudinal gradient. Furthermore, more than 80% of β2-TD was caused by the turnover component (83.4%), while the practically insignificant β2-FD (3.4%) appears mostly due to the nestedness component (Fig 4B).

PERMANOVA showed that dung beetle community composition varies along the altitudinal gradient and that this variation was correlated with climatic, vegetation and soil variables (i.e., the environment) (Table 1).

**Table 1:** Results of PERMANOVA with dung beetles communities along an altitudinal gradient at Serra do Cipó, State of Minas Gerais, Brazil.

**Variables**	**df**	**SS**	**MS**	**F value**	**R**^**2**^	**p value**
**Model 1 –with Altitude only**						
Altitude	1	0.8685	0.86853	2.8747	0.13142	0.003
Residuals	19	5.7405	0.30213			
Total	20	6.6090			1	
**Variables**	**df**	**SS**	**MS**	**F value**	**R**^**2**^	**p value**
**Model 2 –with environmental variables**						
ClimaVeg1	1	0.9775	0.97755	3.7293	0.14791	0.001
ClimaVeg 2	1	0.6637	0.66368	2.5319	0.10042	0.006
Soil 1	1	0.5117	0.51168	1.9520	0.07742	0.023
Residuals	17	4.4561	0.26212			
Total	20	6.6090			1	

df = Degrees of Freedom; SS = Sums of Squares; MS = Mean Squares; F value; R²; and p values. ClimaVeg = Climatic Vegetation Axis of PCA that summarized various climatic and vegetation variables (S1 Appendix).

**Fig 5:**
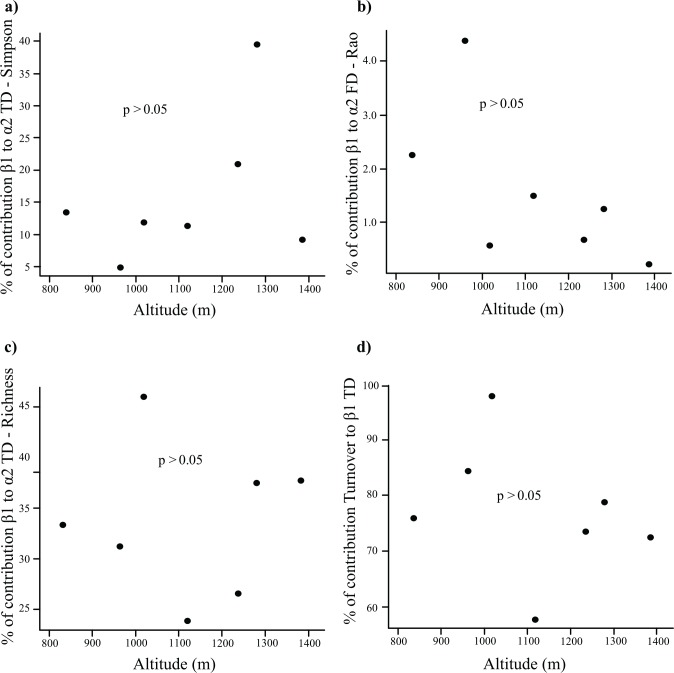
a), b) and c) show the percentage of contribution of the β1 component to α2-TD/FD at each altitude along the gradient. d) Presents the percentage of contribution of the turnover component to β1-TD based on richness at each altitude along the gradient; the values were obtained after the complimentary analysis of multiple-site similarity.

Taxonomic (both richness and Simpson) and functional β1 (diversity among transects of each altitude)contribution to α2 diversity (diversity of each altitude)did not correlate with altitude (Fig 5A, 5B and 5C) (F and P values in S3 Appendix). Likewise, β1-TD and β1-FD were not influenced by any of the measured environmental variables. Besides, altitude and the environmental variables also did not influence the contribution of the turnover component to β1-TD (Fig 5D). We did not perform this last analysis with FD, because the β1 component was very low (Fig 4A).

The analyses of null models showed that taxonomic diversities (TD, spatial levels) were different from what was expected by chance in both null models (presence-absence and abundances matrices; Tables D and E in S3 Appendix). Mean taxonomic diversity of each altitude (α2 diversity) was lower than expected by chance and β2 diversity (diversity among altitudes) was higher. We found different results for functional diversity (FD). When we compared the observed values of FD with the expected by chance values from the null model that considered only species richness the values were similar (Table D in S3 Appendix). However, when we considered abundances to calculate FD, the observed values of FD were lower than expected by chance, except for β2 that was higher (Table E in S3 Appendix). Even in this second case when we used the dung beetles species abundances, the differences of observed to expected values were higher for TD than for FD (Z values in Table E, S3 Appendix).

## Discussion

### Dung beetles taxonomic and functional α diversity

Although the altitudinal range at Serra do Cipó is relatively small, dung beetle richness decreased with altitude, as expected from previous studies (e.g., [38–40]). Richness of dung beetles declined with decreasing temperature and increasing humidity, suggesting that there are fewer species that can survive in the colder and very humid highlands. This may be due to Scarabaeinae being a monophyletic group comprised of warm-adapted species [36], and/or dung beetles feeding mostly on mammal dung, and mammal richness also diminishes with increasing elevation [59,60], thereby reducing food availability. Dung beetle species richness was also positively correlated with abundance, richness and basal area of plants. Vegetation parameters, which also decline with increasing altitude [49], can affect dung beetles directly by acting as a regulator of microclimatic conditions, or indirectly by affecting the vertebrate fauna and consequently food availability [61,62].

When we use the Simpson Index, and thus including abundance, to obtain α-TD, the pattern of decreasing diversity with elevation disappeared, because abundance did not decline with altitude. This result was not expected, but it is due to the presence of dominant species that are well adapted to each altitude and have attained higher population sizes. At all altitudes, the first and second most abundant species together were always much more abundant than the third (at least three times). Contrary to what was also expected, α-FD did not diminish with altitude and its variation was not explained by environmental factors. Actually, the low values of α-FD deserve a little attention (all very close to 1, the minimum possible value). Rao’s Index is maximal when all the species have the same relative abundance and thus maximally functionally dissimilar to each other [24]. Hence, the low values found here means that the most abundant species in a plot are functionally close. Villéger et al. [15] found similar results with estuarine fish communities, and in that case there were functionally close generalist fishes dominating each plot. Here, we found that the most abundant functional group was small paracoprids, which dominate the communities at all altitudes, except for the lowest (800 m a.s.l.).

### Dung beetles taxonomic and functional β diversity

Dung beetle β2 taxonomic diversity (TD; using abundance or not) contributed heavily to γ TD of the entire altitudinal gradient at Serra do Cipó (Fig 4A). Over 50% of the dung beetle γ diversity was caused by differences among altitudes communities, which were influenced by climatic, vegetation and soil variables of the altitudinal gradient, corroborating the hypotheses of environmental filtering [18,63]. The insignificant contribution of β2 functional diversity (FD) to γ FD, and the contrast between β2-TD and β2-FD, showed only small differences in FD among communities, suggesting ecological redundancy [15,22]. In other words, there are different species doing the same “job” (having the same functional traits) along the gradient. In this case, environmental variables are filtering species in terms of the Grinnellian niche, but not in terms of the Eltonian niche [64]. This result is corroborated by the correlation between environmental variables and TD (richness) and species composition, despite there being no correlation between the same variables and FD. Also, the results of null model analysis have brought even more support to this explanation, as it has showed that β2-TD (taxonomic diversity among altitudes) is higher than expected by chance, and that the effect of altitude is influencing both richness and evenness. In the case of FD, null model analysis showed that altitude has effect only in functional identities’ abundances, influencing the evenness of functional groups, as the expected values of FD of the null model that considered only species richness were similar to observed values. The scale of the environmental filter in this study has different impacts on different facets of diversity, such as the local filter being important for TD, but with low impact on FD [14]. Our results reinforce the importance of measuring different facets of diversity in order to understand spatial patterns of biodiversity along environmental gradients [11,25].

Turnover was the main component of β2-TD with a contribution of 83.4% (Fig 4B). This means that highland communities are not sub-sets of lowland communities, but instead they are communities with different species compositions. This finding provides more support for the hypothesis that different environmental filters provided by elevation, select species in terms of the physiological niche (Grinnellian specialization) [64], although it could also arise from dispersal limitations. This also suggests that specialization for surviving at high altitudes results in the loss of competition power at lowland areas [38–40].

Contribution of β1 taxonomic (TD) and β functional (FD) diversity to α2 diversity did not decrease with altitude as we expected (Fig 5A, 5B and 5C). This means that the communities are not becoming more homogeneous with increasing elevation (both in terms of TD and FD). Further, these parameters were not correlated with the environmental variables. We postulate that β diversity within the same altitude is caused by habitat heterogeneity [18,25]. The Serra do Cipó is well known for its landscape heterogeneity, and even at the same altitude we can find several types of habitats associated with different soils [44,65]. Apparently there are different non-nested sub-communities of dung beetles at the same altitudes since turnover is the main component of β1-TD among transects (Fig 5D).

Some consideration to climatic change and dung beetle communities deserves further attention. Tropical insects are particularly sensitive to climatic changes [66] and mountain insects are very vulnerable to global warming [67]. In response to global warming, many species of plants and animals have shifted their altitudinal range upward [10,68,69]. This upslope displacement will lead to serious conservation problems such as extinctions of mountaintop species [70] and lowland biotic attrition as lowland species will migrate upslope [68]. As shown by Larsen [39], Andean dung beetle species occurred upslope in a hotter deforested landscape than in a forested landscape, when the temperature difference between forested and deforested sites was equivalent to 60–100 yr of predicted global warming. Our study showed that more than 50% of dung beetle diversity at Serra do Cipó is due to differences among communities of different altitudes (β2-TD) and these differences are due to almost completely different community compositions (high β2-TD Turnover). Upslope range shifts, and mountaintop and lowland extinctions will lead to even greater loss of taxonomic diversity than expected as diversity among altitudes is high. Although functional diversity did not change with altitude, we do not know how the displacement of species will directly affect dung beetle functions.

## Conclusions

Our study showed that assessing multiple facets of diversity is very informative about how the environment affects communities. Furthermore, partitioning diversity into its spatial components improved our understanding of species distribution and merging these two approaches allowed us to understand the scale of environmental filtering on taxonomic and functional diversity. We showed that there is an ecological redundancy among communities of dung beetles in our altitudinal gradient and environmental variables are filtering species more in terms of the Grinnellian niche and less in terms of the Eltonian niche. Although richness declined with altitude, α-FD did not show this pattern. We found low values of α-FD, indicating that the most abundant species at an elevation are functionally close. Here, the use of different metrics of diversity led to different results, so it is important to know the appropriate metric to use to answer specific questions. The use of Rao index and its decomposition has been shown to be very useful for comparing taxonomic and functional facets of diversity at different spatial scales. Our findings indicated that global warming can bring even bigger losses of diversity than was previously expected, because diversity among altitudes is responsible for at least 50% of regional dung beetle diversity.

## Supporting Information

S1 Appendix Detailed description of principal component analysis. (DOCX)

S2 Appendix List of species and their abundance, functional guild and mean biomass captured in all sampling points. (DOCX)

S3 Appendix Detailed results of all Generalized Linear Models performed in the study and null model analyses. (DOCX)

S4 Appendix Description of the spatial patterns of diversity in rainy and dry seasons. (DOCX)
