# Supplementary material for: Dung Beetles along a Tropical Altitudinal Gradient: Environmental Filtering on Taxonomic and Functional Diversity
Source: PLoS One. 2016 Jun 23;11(6):e0157442. doi: 10.1371/journal.pone.0157442 (PMC4918931; doi:10.1371/journal.pone.0157442)
Supplement: S1 Appendix — (DOCX) [file pone.0157442.s001.docx]

S1 APPENDIX. Detailed description of principal component analysis

Principal component analysis (PCA) on PAST 2.17 program [1] to summarize environmental variables. For all analysis we used the Correlation method to do the PCA.

**A.1 Climatic and vegetation variables**

We use ten variables from the meteorological monitoring tower (equipped with the Onset HOBO^®^ U30 data-logger) to get the climatic scenario of each altitude (September 2013 to June 2014 measurements). They were: means of air temperature, air humidity, soil humidity and photosynthetically active radiation (PAR); variation of air temperature and air humidity; maximum of air temperature; minimum of air temperature and air humidity; and sum of precipitation (Table A). The variation of air temperature and air humidity was calculated with the Coefficient of Variation (C_v_), which is the standard deviation/mean. All calculations were implemented in Microsoft Excel.

TABLE A. Climatic variables used in PCA. Units: Altitude: m a.s.l.; Air Temperature: °C; Air Humidity: relative %; Soil Humidity: m³/m³; PAR:µE; Precipitation: mm.

| Altitude | Mean Temperature | Mean Air Humidity | Mean Soil Humidity | Mean PAR | Temperature variation |
| --- | --- | --- | --- | --- | --- |
| 800 | 21.95 | 73.89 | 0.069 | 442.04 | 0.161 |
| 900 | 21.00 | 76.15 | 0.080 | 408.74 | 0.154 |
| 1000 | 20.48 | 77.60 | 0.201 | 398.79 | 0.154 |
| 1100 | 19.51 | 80.82 | 0.063 | 376.44 | 0.159 |
| 1200 | 19.29 | 81.73 | 0.168 | 420.00 | 0.157 |
| 1300 | 18.40 | 84.00 | 0.087 | 401.86 | 0.157 |
| 1400 | 18.44 | 90.76 | 0.113 | 318.01 | 0.139 |
| Altitude | Air Humidity Variation | Maximum Temperature | Minimum Temperature | Minimum Air Humidity | Precipitation |
| 800 | 0.183 | 35.79 | 9.55 | 25.0 | 780.79 |
| 900 | 0.170 | 34.83 | 11.68 | 26.2 | 640.05 |
| 1000 | 0.164 | 33.73 | 11.05 | 27.3 | 639.34 |
| 1100 | 0.156 | 33.44 | 10.66 | 27.3 | 585.87 |
| 1200 | 0.152 | 32.92 | 11.20 | 28.9 | 670.28 |
| 1300 | 0.149 | 30.95 | 10.83 | 30.3 | 648.12 |
| 1400 | 0.120 | 27.01 | 11.20 | 34.2 | 801.48 |

We used four vegetation variables from Mota (2012), which is also part of “Projeto Ecológico de Longa Duração – Sítio Serra do Cipó”. They were: abundance, richness, height and basal area of plants (Table B). Abundance, richness and basal area were obtained by adding all sampling plots of each altitude and height was the mean value of each altitude. The sampling points totaled 1300 m² per altitude [2].

TABLE B. Vegetation variables used in PCA. Units: altitude: a.s.l.; height: cm; basal area: m²/ha.

| Altitude | Abundance | Richness | Height | Basal Area |
| --- | --- | --- | --- | --- |
| 800 | 1683 | 117 | 117.3 | 6.98 |
| 900 | 1422 | 119 | 110.3 | 2.79 |
| 1000 | 1201 | 78 | 81.79 | 2.51 |
| 1100 | 1456 | 84 | 71.46 | 3.5 |
| 1200 | 1479 | 94 | 60.95 | 2.63 |
| 1300 | 1110 | 48 | 23.81 | 3.09 |
| 1400 | 1421 | 29 | 20.51 | 4.11 |

Because the vegetation variables were correlated with some climatic factors, we performed a PCA with these two groups of environmental variables together. Results of the PCA using the climatic and vegetation variables are summarized in Table C and Fig A.

TABLE C. PCA result with the Eigenvalue and % of variance of each axis (PC).

| PC | Eigenvalue | % variance |
| --- | --- | --- |
| 1 | 8.48905 | 60.636 |
| 2 | 2.83205 | 20.229 |
| 3 | 1.15452 | 8.246 |
| 4 | 0.86552 | 6.182 |
| 5 | 0.39971 | 2.855 |
| 6 | 0.25913 | 1.851 |


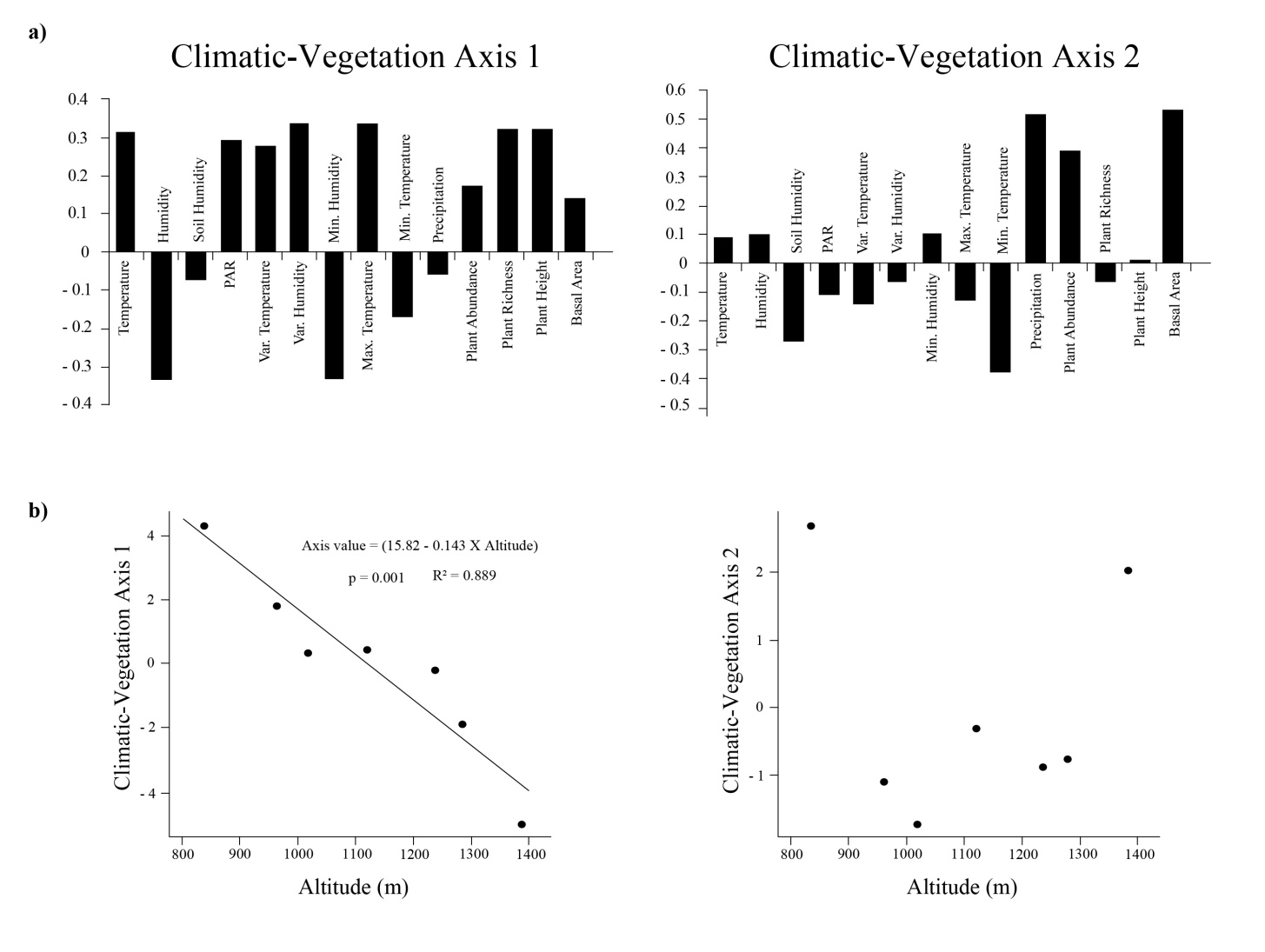


**Fig A.** a) Loadings (Coefficients) of climatic-vegetation axes 1 and 2. b) Climatic-vegetation axes values in each altitude.

Axis 1 can be interpreted as a thermal-humidity-vegetation axis where temperature and vegetation variables are positively correlated and humidity variables negatively correlated. Furthermore, there is a negative correlation between Axis 1 and altitude, showing that temperature variables, as well as vegetation variables, decrease with altitude and humidity variables increase with elevation. Axis 2 can be interpreted most as a precipitation-vegetation axis, but the soil humidity and minimum temperature also influences it.

**A.2 Soil variables**

We used one chemical and four physical soil variables from [3], which is part of “Projeto Ecológico de Longa Duração – Sítio Serra do Cipó” as well as this study. They were: chemical: organic matter; physicals: coarse sand, fine sand, silt and clay (Table D).

TABLE D. Soil variables used in PCA. Units: altitude: m a.s.l; all others: dag/Kg.

| Altitude | Organic matter | Coarse Sand | Fine Sand | Silt | Clay |
| --- | --- | --- | --- | --- | --- |
| 800 | 3.510 | 17.846 | 62.769 | 11.538 | 7.846 |
| 900 | 5.038 | 4.176 | 72.592 | 12.461 | 10.769 |
| 1000 | 3.574 | 17.653 | 67.115 | 8.153 | 7.076 |
| 1100 | 5.593 | 4.876 | 81.123 | 5.538 | 8.461 |
| 1200 | 6.899 | 12.800 | 75.661 | 6.000 | 5.538 |
| 1300 | 3.555 | 18.141 | 73.691 | 5.000 | 3.166 |
| 1400 | 6.901 | 9.353 | 78.492 | 7.846 | 4.307 |

Results of the PCA using the soil variables are summarized in Table E and Fig B.

TABLE E. PCA result with the Eigenvalue and % of variance of each axis (PC).

| PC | Eigenvalue | % variance |
| --- | --- | --- |
| 1 | 2.49068 | 49.814 |
| 2 | 1.95573 | 39.115 |
| 3 | 0.427345 | 8.5469 |
| 4 | 0.126244 | 2.5249 |
| 5 | 1.11E-13 | 2.22E-12 |

Axis 1 represents soils that have high values of organic matter and fine sand but little values of coarse sand, silt and clay (sandy soils). In the contrary, Axis 2 represents soils that have high values of silt and clay (clayey soils). The composition of soils can change the soil retention capacity and in this case, Axis 2 can be interpreted as soils that retain much water.


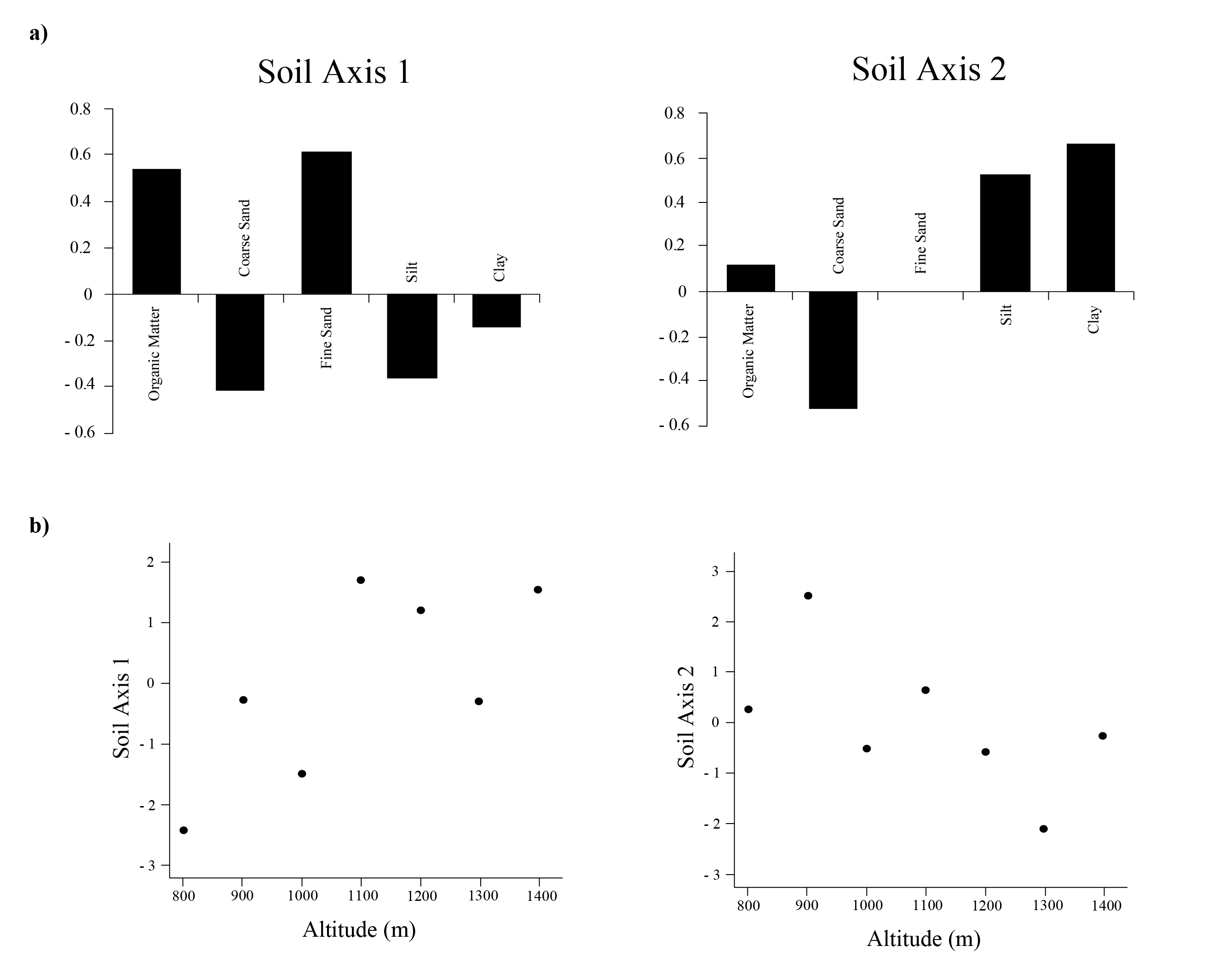


**Fig B.** a) Loadings (Coefficients) of soil axes 1 and 2. b) Soil axes values in each altitude.

LITERATURE CITED

1. Hammer Ø, Harper DAT, Ryan PD. Paleontological statistics software package for education and data analysis. Palaeontol Electron. 2001;4: 9–18. doi:10.1016/j.bcp.2008.05.025

2. Mota GS. Variação na estrutura, na composição florística e nas formas de vida ao longo de um gradiente altitudinal na Cadeia do Espinhaço. Universidade Estadual de Montes Claros. 2012.

3. Coutinho ES, Fernandes GW, Berbara RLL, Valério HM, Goto BT. Variation of arbuscular mycorrhizal fungal communities along an altitudinal gradient in rupestrian grasslands in Brazil. Mycorrhiza. 2015; doi:10.1007/s00572-015-0636-5
