# Supplementary material for: Dung Beetles along a Tropical Altitudinal Gradient: Environmental Filtering on Taxonomic and Functional Diversity
Source: PLoS One. 2016 Jun 23;11(6):e0157442. doi: 10.1371/journal.pone.0157442 (PMC4918931; doi:10.1371/journal.pone.0157442)
Supplement: S2 Appendix — (DOCX) [file pone.0157442.s002.docx]

S2 APPENDIX. List of species and their abundance, functional guild and mean biomass captured in all sampling points.

TABLE A. Number of individuals, by species, captured in all transects and functional guild and mean biomass of each species.

| **Species** | **Functional Guild** | **Mean Biomass (mg)** | **Number of Individuals** | | | | | | | | | | | | | | | | | | | | |
| --- | --- | --- | --- | --- | --- | --- | --- | --- | --- | --- | --- | --- | --- | --- | --- | --- | --- | --- | --- | --- | --- | --- | --- |
| Altitude (m a.s.l.) | | | 1400 | | | 1300 | | | 1200 | | | 1100 | | | 1000 | | | 900 | | | 800 | | |
| Transects | | | I | II | III | I | II | III | I | II | III | I | II | III | I | II | III | I | II | III | I | II | III |
| *Agamopus unguicularis* | Paracoprid | 3.8 | 0 | 0 | 0 | 0 | 0 | 0 | 0 | 0 | 0 | 0 | 0 | 0 | 1 | 0 | 0 | 0 | 0 | 0 | 0 | 0 | 1 |
| *Agamopus viridis* | Paracoprid | 3.0 | 0 | 0 | 0 | 0 | 1 | 0 | 1 | 0 | 0 | 0 | 0 | 0 | 0 | 1 | 2 | 0 | 0 | 1 | 1 | 1 | 0 |
| *Anomiopus virescens* | Paracoprid | 11.2 | 0 | 0 | 0 | 0 | 0 | 1 | 0 | 0 | 0 | 0 | 0 | 0 | 0 | 0 | 0 | 0 | 1 | 0 | 0 | 0 | 0 |
| *Ateuchus vividus* | Paracoprid | 21.7 | 1 | 0 | 0 | 0 | 0 | 0 | 0 | 0 | 0 | 1 | 0 | 1 | 15 | 6 | 8 | 1 | 0 | 0 | 0 | 0 | 0 |
| *Canthidium aff barbacenicum* | Paracoprid | 8.6 | 0 | 0 | 0 | 0 | 0 | 0 | 0 | 0 | 0 | 2 | 0 | 0 | 0 | 0 | 0 | 0 | 0 | 0 | 4 | 3 | 4 |
| *Canthidium sp 1* | Paracoprid | 6.8 | 1 | 0 | 0 | 1 | 15 | 2 | 25 | 12 | 11 | 10 | 16 | 4 | 1 | 0 | 2 | 24 | 4 | 9 | 32 | 14 | 12 |
| *Canthidium sp 2* | Paracoprid | 8.3 | 0 | 0 | 0 | 0 | 0 | 0 | 0 | 0 | 0 | 0 | 0 | 0 | 0 | 0 | 0 | 0 | 0 | 2 | 0 | 0 | 0 |
| *Canthidium sp 3* | Paracoprid | 11.2 | 0 | 0 | 0 | 0 | 1 | 0 | 5 | 3 | 3 | 2 | 2 | 1 | 0 | 0 | 0 | 3 | 0 | 1 | 25 | 19 | 9 |
| *Canthidium sp 4* | Paracoprid | 8.2 | 0 | 0 | 0 | 0 | 0 | 0 | 0 | 0 | 0 | 1 | 0 | 5 | 0 | 0 | 1 | 0 | 1 | 0 | 0 | 0 | 0 |
| *Canthidium sp 5* | Paracoprid | 7.2 | 2 | 11 | 1 | 0 | 0 | 0 | 0 | 0 | 0 | 0 | 0 | 0 | 0 | 0 | 0 | 0 | 0 | 0 | 0 | 0 | 0 |
| *Canthidium sp 6* | Paracoprid | 11.8 | 1 | 1 | 8 | 0 | 0 | 0 | 0 | 1 | 0 | 0 | 0 | 0 | 0 | 0 | 0 | 1 | 0 | 0 | 0 | 0 | 0 |
| *Canthidium sp 8* | Paracoprid | 7.9 | 0 | 0 | 0 | 7 | 0 | 4 | 1 | 0 | 1 | 7 | 4 | 9 | 9 | 0 | 5 | 1 | 3 | 3 | 0 | 0 | 0 |
| *Canthidium sp 10* | Paracoprid | 14.7 | 0 | 0 | 0 | 0 | 0 | 0 | 1 | 0 | 0 | 0 | 0 | 0 | 0 | 0 | 0 | 17 | 8 | 31 | 21 | 0 | 2 |
| *Canthon aff podagricus* | Telecoprid | 6.4 | 0 | 0 | 0 | 0 | 0 | 0 | 0 | 0 | 0 | 0 | 0 | 3 | 0 | 0 | 0 | 0 | 0 | 0 | 0 | 0 | 0 |
| *Canthon lamproderes* | Telecoprid | 72.9 | 0 | 0 | 0 | 0 | 0 | 0 | 0 | 0 | 0 | 0 | 0 | 0 | 0 | 0 | 0 | 0 | 0 | 0 | 10 | 29 | 2 |
| *Canthon sp 1* | Telecoprid | 5.9 | 0 | 0 | 0 | 16 | 77 | 3 | 42 | 21 | 9 | 11 | 81 | 9 | 0 | 0 | 9 | 59 | 113 | 7 | 0 | 25 | 0 |
| *Canthon sp 2* | Telecoprid | 30.0 | 0 | 0 | 0 | 0 | 0 | 0 | 0 | 0 | 0 | 0 | 0 | 0 | 0 | 0 | 0 | 0 | 0 | 0 | 0 | 0 | 4 |
| *Canthon sp 3* | Telecoprid | 18.4 | 0 | 0 | 0 | 0 | 1 | 0 | 0 | 0 | 0 | 0 | 0 | 0 | 0 | 0 | 0 | 0 | 0 | 0 | 3 | 3 | 4 |
| *Canthon unicolor* | Telecoprid | 45.3 | 0 | 0 | 0 | 0 | 0 | 0 | 0 | 0 | 0 | 0 | 0 | 0 | 0 | 0 | 0 | 0 | 0 | 0 | 2 | 14 | 1 |
| *Coprophanaeus cyanescens* | Paracoprid | 690.8 | 0 | 0 | 0 | 0 | 0 | 0 | 0 | 0 | 0 | 0 | 0 | 0 | 0 | 0 | 0 | 0 | 0 | 0 | 0 | 0 | 1 |
| *Coprophanaeus ensifer* | Paracoprid | 2050.0 | 0 | 0 | 0 | 0 | 0 | 0 | 0 | 0 | 0 | 0 | 0 | 0 | 0 | 0 | 0 | 0 | 0 | 0 | 1 | 0 | 0 |
| *Coprophanaeus jasius* | Paracoprid | 894.7 | 0 | 0 | 0 | 0 | 0 | 0 | 0 | 0 | 0 | 0 | 0 | 0 | 0 | 0 | 0 | 0 | 0 | 0 | 0 | 0 | 1 |
| *Coprophanaeus milon magnoi* | Paracoprid | 751.0 | 0 | 0 | 0 | 0 | 0 | 0 | 1 | 0 | 0 | 2 | 0 | 1 | 0 | 0 | 2 | 0 | 0 | 0 | 0 | 0 | 3 |
| *Deltochilum inaequale* | Telecoprid | 582.3 | 5 | 3 | 0 | 0 | 0 | 6 | 6 | 0 | 4 | 0 | 0 | 0 | 0 | 0 | 1 | 0 | 0 | 0 | 0 | 0 | 0 |
| *Deltochilum komareki* | Telecoprid | 74.4 | 0 | 0 | 0 | 0 | 0 | 0 | 0 | 0 | 0 | 0 | 0 | 0 | 0 | 0 | 0 | 1 | 1 | 3 | 2 | 4 | 2 |
| *Deltochilum pseudoicarus* | Telecoprid | 464.2 | 0 | 0 | 0 | 6 | 18 | 0 | 0 | 1 | 0 | 1 | 3 | 2 | 0 | 0 | 1 | 6 | 7 | 1 | 0 | 1 | 0 |
| *Dichotomius bos* | Paracoprid | 347.7 | 0 | 0 | 0 | 0 | 0 | 0 | 0 | 0 | 0 | 0 | 0 | 0 | 0 | 1 | 0 | 0 | 0 | 0 | 0 | 3 | 3 |
| *Dichotomius crinicollis* | Paracoprid | 385.2 | 0 | 0 | 0 | 0 | 0 | 0 | 0 | 0 | 0 | 0 | 0 | 0 | 1 | 0 | 0 | 0 | 0 | 0 | 1 | 0 | 4 |
| *Dichotomius depressicollis* | Paracoprid | 338.3 | 0 | 1 | 0 | 2 | 7 | 3 | 9 | 0 | 25 | 8 | 7 | 8 | 20 | 10 | 13 | 49 | 41 | 31 | 33 | 46 | 13 |
| *Dichotomius glaucus* | Paracoprid | 289.0 | 0 | 0 | 0 | 0 | 1 | 0 | 0 | 0 | 0 | 3 | 1 | 0 | 1 | 0 | 0 | 0 | 0 | 0 | 0 | 0 | 0 |
| *Dichotomius luctuosus* | Paracoprid | 197.3 | 0 | 7 | 3 | 0 | 0 | 0 | 1 | 1 | 1 | 0 | 0 | 0 | 0 | 2 | 0 | 0 | 0 | 1 | 0 | 0 | 1 |
| *Dichotomius nisus* | Paracoprid | 309.8 | 0 | 0 | 0 | 0 | 1 | 1 | 0 | 0 | 0 | 2 | 0 | 1 | 2 | 2 | 5 | 1 | 0 | 1 | 1 | 1 | 1 |
| *Digitonthophagus gazella* | Paracoprid | 51.0 | 0 | 0 | 0 | 0 | 0 | 0 | 0 | 0 | 0 | 0 | 0 | 1 | 0 | 1 | 0 | 0 | 0 | 0 | 0 | 0 | 0 |
| *Eurysternus nigrovirens* | Endocoprid | 8.7 | 0 | 2 | 0 | 0 | 5 | 2 | 24 | 30 | 6 | 10 | 8 | 2 | 1 | 0 | 0 | 4 | 3 | 1 | 2 | 1 | 2 |
| *Genieridium bidens* | Endocoprid | 6.6 | 0 | 0 | 0 | 0 | 0 | 0 | 1 | 0 | 1 | 0 | 0 | 0 | 0 | 1 | 0 | 0 | 1 | 0 | 0 | 0 | 1 |
| *Genieridium cryptops* | Endocoprid | 6.0 | 0 | 0 | 0 | 0 | 0 | 0 | 0 | 0 | 0 | 0 | 0 | 0 | 0 | 0 | 0 | 0 | 0 | 0 | 1 | 0 | 0 |
| *Homocopris sp 1* | Paracoprid | 79.2 | 0 | 0 | 0 | 0 | 0 | 0 | 6 | 4 | 1 | 0 | 0 | 0 | 1 | 0 | 0 | 3 | 0 | 2 | 0 | 0 | 0 |
| *Homocopris sp 2* | Paracoprid | 40.4 | 0 | 0 | 0 | 0 | 0 | 0 | 0 | 7 | 0 | 0 | 0 | 0 | 0 | 0 | 0 | 0 | 0 | 0 | 0 | 0 | 0 |
| *Homocopris sp 3* | Paracoprid | 69.0 | 0 | 0 | 0 | 0 | 0 | 0 | 0 | 4 | 0 | 0 | 0 | 0 | 0 | 0 | 0 | 0 | 0 | 0 | 0 | 0 | 0 |
| *Ontherus (Caelontherus) sp* | Paracoprid | 26.0 | 0 | 0 | 0 | 0 | 1 | 0 | 0 | 0 | 0 | 0 | 0 | 0 | 0 | 0 | 0 | 0 | 0 | 0 | 0 | 0 | 0 |
| *Ontherus aff carinifrons* | Paracoprid | 39.6 | 0 | 0 | 1 | 1 | 2 | 1 | 1 | 2 | 4 | 0 | 2 | 0 | 0 | 0 | 0 | 1 | 0 | 0 | 0 | 0 | 0 |
| *Ontherus appendiculatus* | Paracoprid | 58.2 | 0 | 0 | 0 | 0 | 0 | 0 | 0 | 0 | 0 | 0 | 0 | 0 | 7 | 2 | 1 | 0 | 1 | 1 | 2 | 2 | 2 |
| *Onthophagus aff buculus* | Paracoprid | 11.1 | 2 | 1 | 2 | 0 | 0 | 0 | 1 | 0 | 0 | 0 | 0 | 1 | 1 | 0 | 0 | 1 | 0 | 1 | 2 | 0 | 0 |
| *Onthophagus aff hirculus* | Paracoprid | 9.7 | 188 | 31 | 43 | 13 | 12 | 16 | 20 | 5 | 10 | 9 | 28 | 17 | 28 | 19 | 27 | 7 | 17 | 15 | 15 | 13 | 16 |
| *Oxysternon palaemon* | Paracoprid | 273.4 | 0 | 0 | 0 | 1 | 1 | 0 | 0 | 0 | 0 | 0 | 0 | 0 | 7 | 1 | 1 | 11 | 3 | 2 | 133 | 28 | 44 |
| *Phanaeus dzidoi* | Paracoprid | 130.0 | 0 | 0 | 0 | 0 | 0 | 0 | 0 | 0 | 0 | 0 | 0 | 0 | 0 | 0 | 0 | 0 | 0 | 0 | 1 | 0 | 1 |
| *Phanaeus kirbyi ledezmai* | Paracoprid | 202.0 | 0 | 0 | 0 | 0 | 0 | 1 | 0 | 0 | 0 | 0 | 0 | 0 | 0 | 0 | 0 | 0 | 0 | 0 | 0 | 0 | 0 |
| *Phanaeus palaeno* | Paracoprid | 215.3 | 0 | 0 | 0 | 0 | 0 | 3 | 0 | 0 | 0 | 1 | 0 | 0 | 1 | 0 | 0 | 0 | 0 | 0 | 17 | 0 | 5 |
| *Sulcophanaeus menelas* | Paracoprid | 428.3 | 47 | 30 | 19 | 5 | 3 | 11 | 7 | 5 | 6 | 3 | 3 | 12 | 0 | 2 | 4 | 1 | 1 | 0 | 0 | 0 | 0 |
| *Trichillum sp 1* | Endocoprid | 0.7 | 0 | 0 | 0 | 1 | 27 | 0 | 10 | 33 | 5 | 1 | 2 | 1 | 1 | 1 | 5 | 31 | 18 | 8 | 4 | 3 | 9 |
| *Trichillum sp 2* | Endocoprid | 0.9 | 0 | 1 | 0 | 0 | 0 | 0 | 0 | 0 | 0 | 0 | 0 | 0 | 0 | 4 | 0 | 0 | 2 | 0 | 0 | 0 | 0 |
| *Uroxys sp 1* | Paracoprid | 1.1 | 0 | 0 | 0 | 25 | 92 | 0 | 97 | 79 | 13 | 11 | 39 | 8 | 0 | 1 | 0 | 30 | 14 | 8 | 0 | 1 | 0 |
| *Uroxys sp 2* | Paracoprid | 0.6 | 0 | 2 | 2 | 2 | 40 | 0 | 43 | 35 | 11 | 5 | 6 | 2 | 0 | 0 | 0 | 12 | 5 | 10 | 19 | 2 | 1 |
| *Uroxys sp 3* | Paracoprid | 1.5 | 0 | 0 | 0 | 16 | 27 | 0 | 49 | 32 | 3 | 4 | 32 | 3 | 0 | 0 | 0 | 24 | 0 | 6 | 9 | 0 | 0 |
| *Uroxys sp 4* | Paracoprid | 2.2 | 0 | 0 | 0 | 0 | 0 | 0 | 0 | 0 | 0 | 1 | 0 | 1 | 0 | 0 | 0 | 0 | 0 | 1 | 2 | 0 | 0 |
| *Uroxys sp 5* | Paracoprid | 12.0 | 0 | 1 | 0 | 0 | 0 | 0 | 0 | 0 | 0 | 0 | 0 | 0 | 0 | 0 | 0 | 0 | 0 | 0 | 0 | 0 | 0 |

Figure A presents the abundance ranking of all dung beetle communities recorded with their functional group highlighted. At all altitudes the first and second most abundant species together were at least three times more abundant than the third (at 1400 m a.s.l. they were 25 times!). In three of the seven altitude classes, most abundant species were at least twice more abundant than second. The most abundant functional group was at least twice more abundant than the next most abundant functional group in five of the altitude classes (at 1200 m a.s.l. it was 6 times). Small paracoprids were the dominant functional group at all altitudes, except for the lowest (800 m a.s.l.), where large paracoprids dominated (Fig A).


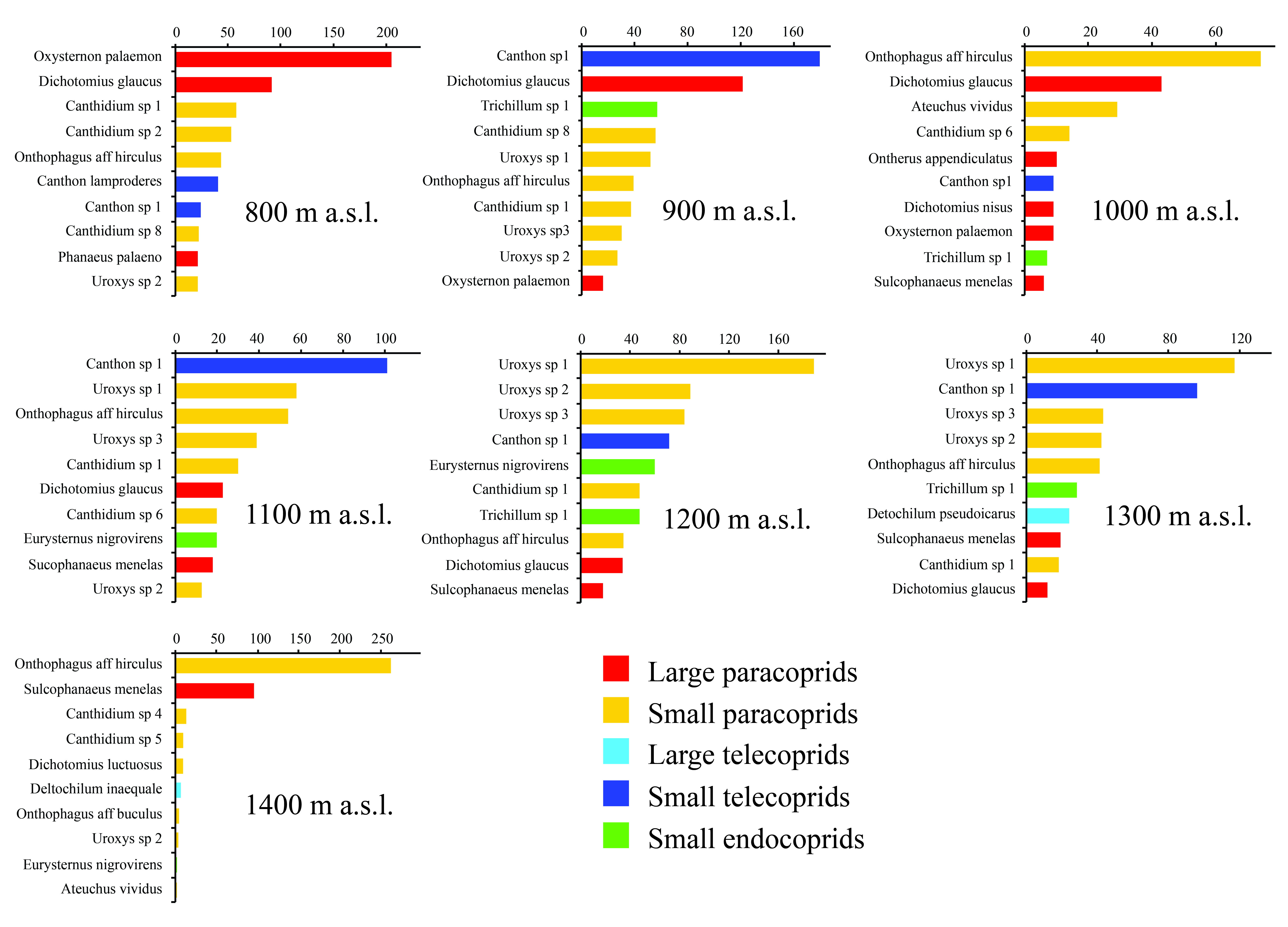


**Fig A.** Abundance rankings of dung beetle communities of all altitudes of the altitudinal gradient at Serra do Cipó, State of Minas Gerais, Brazil. The colors highlight the functional group of each species.
