# Supplementary material for: Dung Beetles along a Tropical Altitudinal Gradient: Environmental Filtering on Taxonomic and Functional Diversity
Source: PLoS One. 2016 Jun 23;11(6):e0157442. doi: 10.1371/journal.pone.0157442 (PMC4918931; doi:10.1371/journal.pone.0157442)
Supplement: S3 Appendix — (DOCX) [file pone.0157442.s003.docx]

S3 APPENDIX. Detailed results of all Generalized Linear Models performed in the study and null model analyses.

TABLE A. Results of GLM’s with abundance, richness and α TD and α FD indices. Each Response Variable in the table represents a model (Abundance/Richness are *Quasipoisson* and α TD/FD are *Gaussian* error distribution). For the significant variables, are presented values of the minimum model and for the non-significant variables, values of the maximum model. D.F.= Degrees of Freedom; Dev.= Deviance; Res. D.F.= Residual Degrees of Freedom; Res. Dev.= Residual Deviance; F= F values.

| Response Variable | Explanatory Variables | D.F. | Dev. | Res. D.F. | Res. Dev. | F | P-value |
| --- | --- | --- | --- | --- | --- | --- | --- |
|  |  |  |  |  |  |  |  |
| Abundance | Altitude | 1 | 26.54 | 5 | 394.96 | 0.365 | 0.572 |
|  |  |  |  |  |  |  |  |
| Abundance | Climatic-vegetation Axis 1 | 1 | 85.81 | 5 | 335.69 | 0.689 | 0.493 |
|  | Climatic-vegetation Axis 2 | 1 | 16.54 | 4 | 319.15 | 0.132 | 0.750 |
|  | Soil Axis 1 | 1 | 55.77 | 3 | 263.37 | 0.448 | 0.572 |
|  | Soil Axis 2 | 1 | 0.45 | 2 | 262.91 | 0.003 | 0.957 |
|  | Null |  |  | 6 | 421.51 | 0.318 | 0.848 |
|  |  |  |  |  |  |  |  |
| Richness | **Altitude** | **1** | **9.405** | **5** | **1.97** | **24.31** | **0.004** |
|  |  |  |  |  |  |  |  |
| Richness | **Climatic-vegetation Axis 1** | **1** | **10.52** | **5** | **0.86** | **61.09** | **0.0005** |
|  | Null |  |  | 6 | 11.38 |  |  |
|  |  |  |  |  |  |  |  |
| α TD – Simpson | Altitude | 1 | 7.56 | 5 | 20.47 | 1.84 | 0.232 |
|  |  |  |  |  |  |  |  |
| α TD – Simpson | Climatic-vegetation Axis 1 | 1 | 15.14 | 5 | 12.88 | 16.64 | 0.055 |
|  | Climatic-vegetation Axis 2 | 1 | 4.72 | 4 | 8.15 | 5.19 | 0.150 |
|  | Soil Axis 1 |  | 1.87 | 3 | 6.28 | 2.06 | 0.287 |
|  | Soil Axis 2 |  | 4.46 | 2 | 1.81 | 4.90 | 0.157 |
|  | Null |  |  | 6 | 28.03 | 7.20 | 0.125 |
|  |  |  |  |  |  |  |  |
| α FD – Rao | Altitude | 1 | 0.005 | 19 | 0.086 | 0.32 | 0.591 |
|  |  |  |  |  |  |  |  |
| α FD – Rao | Climatic-vegetation Axis 1 | 1 | 0.016 | 5 | 0.075 | 0.74 | 0.479 |
|  | Climatic-vegetation Axis 2 | 1 | 0.018 | 4 | 0.057 | 0.80 | 0.464 |
|  | Soil Axis 1 | 1 | 0.011 | 3 | 0.045 | 0.50 | 0.550 |
|  | Soil Axis 2 | 1 | 2e-04 | 2 | 0.045 | 0.01 | 0.308 |
|  | Null |  |  | 6 | 0.092 | 0.51 | 0.926 |

TABLE B. Results of GLM’s with β TD, β FD and TD Turnover. Each Response Variable in the table represents a model with *Gaussian* error distribution. D.F.= Degrees of Freedom; Dev.= Deviance; Res. D.F.= Residual Degrees of Freedom; Res. Dev.= Residual Deviance; F= F values.

| Response Variable | Explanatory Variables | D.F. | Dev. | Res. D.F. | Res. Dev. | F | P-value |
| --- | --- | --- | --- | --- | --- | --- | --- |
|  |  |  |  |  |  |  |  |
| β TD – Simpson | Altitude | 1 | 1.38 | 5 | 524.9 | 0.013 | 0.913 |
|  |  |  |  |  |  |  |  |
| β TD – Simpson | Climatic-vegetation Axis 1 | 1 | 8.20 | 5 | 518.1 | 0.036 | 0.867 |
|  | Climatic-vegetation Axis 1 | 1 | 18.43 | 4 | 499.6 | 0.080 | 0.802 |
|  | Soil Axis 1 | 1 | 9.63 | 3 | 490.0 | 0.042 | 0.856 |
|  | Soil Axis 2 | 1 | 33.67 | 2 | 456.3 | 0.147 | 0.737 |
|  | Null |  | 69.96 | 6 | 526.3 | 0.076 | 0.982 |
|  |  |  |  |  |  |  |  |
| β FD – Rao | Altitude | 1 | 4.91 | 5 | 7.296 | 3.369 | 0.125 |
|  |  |  |  |  |  |  |  |
| β FD – Rao | Climatic-vegetation Axis 1 | 1 | 4.61 | 5 | 7.60 | 2.605 | 0.247 |
|  | Climatic-vegetation Axis 1 | 1 | 0.080 | 4 | 7.52 | 0.045 | 0.850 |
|  | Soil Axis 1 | 1 | 0.169 | 3 | 7.35 | 0.095 | 0.786 |
|  | Soil Axis 2 | 1 | 3.81 | 2 | 3.54 | 2.15 | 0.280 |
|  | Null |  | 8.67 | 6 | 12.21 | 1.22 | 0.495 |
|  |  |  |  |  |  |  |  |
| β TD – Richness | Altitude | 1 | 6e-06 | 5 | 0.033 | 0.001 | 0.975 |
|  |  |  |  |  |  |  |  |
| β TD – Richness | Climatic-vegetation Axis 1 | 1 | 0.002 | 5 | 0.031 | 2.04 | 0.289 |
|  | Climatic-vegetation Axis 1 | 1 | 1e-04 | 4 | 0.030 | 0.14 | 0.739 |
|  | Soil Axis 1 | 1 | 0.028 | 3 | 0.002 | 28.00 | 0.033 |
|  | Soil Axis 2 | 1 | 6e-04 | 2 | 0.002 | 0.65 | 0.504 |
|  | Null |  | 0.031 | 6 | 0.033 | 7.71 | 0.118 |
|  |  |  |  |  |  |  |  |
| Turnover β TD – Richness | Altitude | 1 | 0.008 | 5 | 0.080 | 0.498 | 0.511 |
|  |  |  |  |  |  |  |  |
| Turnover β TD – Richness | Climatic-vegetation Axis 1 | 1 | 0.0015 | 5 | 0.087 | 0.36 | 0.605 |
|  | Climatic-vegetation Axis 1 | 1 | 0.0139 | 4 | 0.073 | 3.31 | 0.210 |
|  | Soil Axis 1 | 1 | 0.0581 | 3 | 0.015 | 13.85 | 0.065 |
|  | Soil Axis 2 | 1 | 0.0069 | 2 | 0.008 | 1.65 | 0.326 |
|  | Null |  | 0.0806 | 6 | 0.089 | 4.79 | 0.179 |

TABLE C. Results of additive partition of dung beetles taxonomic diversity (Richness). The expected diversities in null models were calculated 999 times by individual based randomization of the community data matrix and then compared with the observed values of diversities. Div. = Diversities; Obs. = Observed values; SES = Standardized effect sizes; Mean Sim. = Mean values of the 999 simulations; P = P values.

| **Div.** | **Obs.** | **SES** | **Mean Sim.** | **P** |
| --- | --- | --- | --- | --- |
| **α 1** | 17.52 | -29.97 | 26.83 | **0.001** |
| **β 1** | 8.90 | -5.91 | 12.11 | **0.001** |
| **α 2** | 26.42 | -21.65 | 38.94 | **0.001** |
| **β 2** | 29.57 | 21.65 | 17.05 | **0.001** |
| **γ** | 56.00 | 0.00 | 56.00 | 1.000 |

TABLE D. Results of additive partition of dung beetles taxonomic (Simpson index) and functional (Rao index) diversities. The expected diversities in null models were calculated 999 times by individual based randomization of the community data matrix (presence-absence matrix) and then compared with the observed values of diversities. Div. = Diversities; Obs. = Observed values; SES = Standardized effect sizes (Z values); Mean Sim. = Mean values of the 999 simulations; P = P values.

| **Diversity** | **Obs.** | **SES** | **Mean Sim.** | **P** |
| --- | --- | --- | --- | --- |
| **Taxonomic** |  |  |  |  |
| **α 1** | 15.82 | 0 | 15.82 | 1.000 |
| **β 1** | 8.92 | -6.996 | 13.51 | **0.001** |
| **α 2** | 24.74 | -6.996 | 29.33 | **0.001** |
| **β 2** | 31.25 | 6.996 | 26.66 | **0.001** |
| **γ** | 56 | 0 | 56 | 1.000 |
| **Functional** |  |  |  |  |
| **α 1** | 1.294 | -2.187 | 1.308 | **0.014** |
| **β 1** | 0.020 | 0.435 | 0.016 | 0.331 |
| **α 2** | 1.315 | -0.919 | 1.325 | 0.178 |
| **β 2** | 0.041 | 0.919 | 0.031 | 0.179 |
| **γ** | 1.356 | 0 | 1.356 | 1 |

TABLE E. Results of additive partition of dung beetles taxonomic (Simpson index) and functional (Rao index) diversities. The expected diversities in null models were calculated 999 times by individual based randomization of the community data matrix (count matrix), maintaining richness per transect and number of transects that each species occur. The number of individuals of each transect was also maintained (support capacity). Then, the expected values were compared with the observed values of diversities. Div. = Diversities; Obs. = Observed values; SES = Standardized effect sizes (Z values); Mean Sim. = Mean values of the 999 simulations; P = P values.

| **Div.** | **Obs.** | **SES** | **Mean Sim.** | **P** |
| --- | --- | --- | --- | --- |
| **Taxonomic** |  |  |  |  |
| **α 1** | 5.38 | -73.042 | 14.391 | **0.001** |
| **β 1** | 0.26 | -13.895 | 7.24 | **0.001** |
| **α 2** | 5.64 | -31.919 | 21.63 | **0.001** |
| **β 2** | 6.97 | -4.536 | 10.23 | **0.001** |
| **γ** | 12.62 | -26.528 | 31.87 | **0.001** |
| **Proportional to γ** |  |  |  |  |
| **α 1 prop.** | 42.61 | -2.330 | 45.17 | **0.001** |
| **β 1 prop.** | 2.10 | -14.036 | 22.74 | **0.001** |
| **α 2 prop.** | 44.72 | -13.192 | 67.91 | **0.001** |
| **β 2 prop.** | 55.27 | 13.192 | 32.08 | **0.001** |
| **Functional** |  |  |  |  |
| **α 1** | 1.233 | -6.949 | 1.306 | **0.001** |
| **β 1** | 0.022 | 1.267 | 0.010 | 0.103 |
| **α 2** | 1.256 | -4.254 | 1.316 | **0.001** |
| **β 2** | 0.045 | 5.226 | 0.006 | **0.001** |
| **γ** | 1.302079 | -1.595 | 1.32294 | 0.055 |
| **Proportional to γ** |  |  |  |  |
| **α 1 prop.** | 94.770 | -4.494 | 98.733 | **0.001** |
| **β 1 prop.** | 1.758 | 1.321 | 0.765 | 0.093 |
| **α 2 prop.** | 96.528 | -5.334 | 99.498 | **0.001** |
| **β 2 prop.** | 3.471 | 5.335 | 0.500 | **0.001** |
