# Supplementary material for: Dung Beetles along a Tropical Altitudinal Gradient: Environmental Filtering on Taxonomic and Functional Diversity
Source: PLoS One. 2016 Jun 23;11(6):e0157442. doi: 10.1371/journal.pone.0157442 (PMC4918931; doi:10.1371/journal.pone.0157442)
Supplement: S4 Appendix — (DOCX) [file pone.0157442.s004.docx]

S4 APPENDIX. Description of the spatial patterns of diversity in rainy and dry seasons

The study was conducted along an altitudinal gradient of cerrado and rupestrian grassland areas located in the southern part of the Espinhaço mountain range, in State of Minas Gerais, Brazil (19°10’ and 19°22’ S, 43°29’ and 43°36’ W) between September 2013 and June 2014. Dung beetles were sampled during four time periods of the year: end of dry season (September 2013), beginning of rainy season (December 2013), end of rainy season (March 2014) and beginning of dry season (June 2014). The data from all samples (four periods) of each area were pooled (summed), resulting in one value per altitude (n=7).

Scarabaeinae community composition change along the year, however we show below that the spatial pattern does not, and it would be no problem in pooling the data of an entire year to study the spatial pattern. We used data from rainy and dry season separately to perform the same analysis that are presented in the paper. Data collected in December 2013 and March 2014 are similar in terms of species composition because they are from rainy season and data collected in September 2013 and June 2014 are similar because they are from dry season. We did a Non Metric Multidimensional Scaling analysis (NMDS) to show that the communities of the two seasons are indeed very different in composition (Fig A). However, the variation of abundance and richness with altitude remains equal to the pattern found when we pooled the data (Fig B). In the dry season there are fewer species than in the rainy season, but the decreasing in species richness with altitude increase still remains. The partition of diversity showed the same trend (Fig C). Taxonomic beta diversity is higher than expected by chance and higher than functional beta diversity in both seasons (Table A). The climatic pattern also remains the same along the year, as showed by Principal Component Analysis (PCA) for both seasons (Fig D).


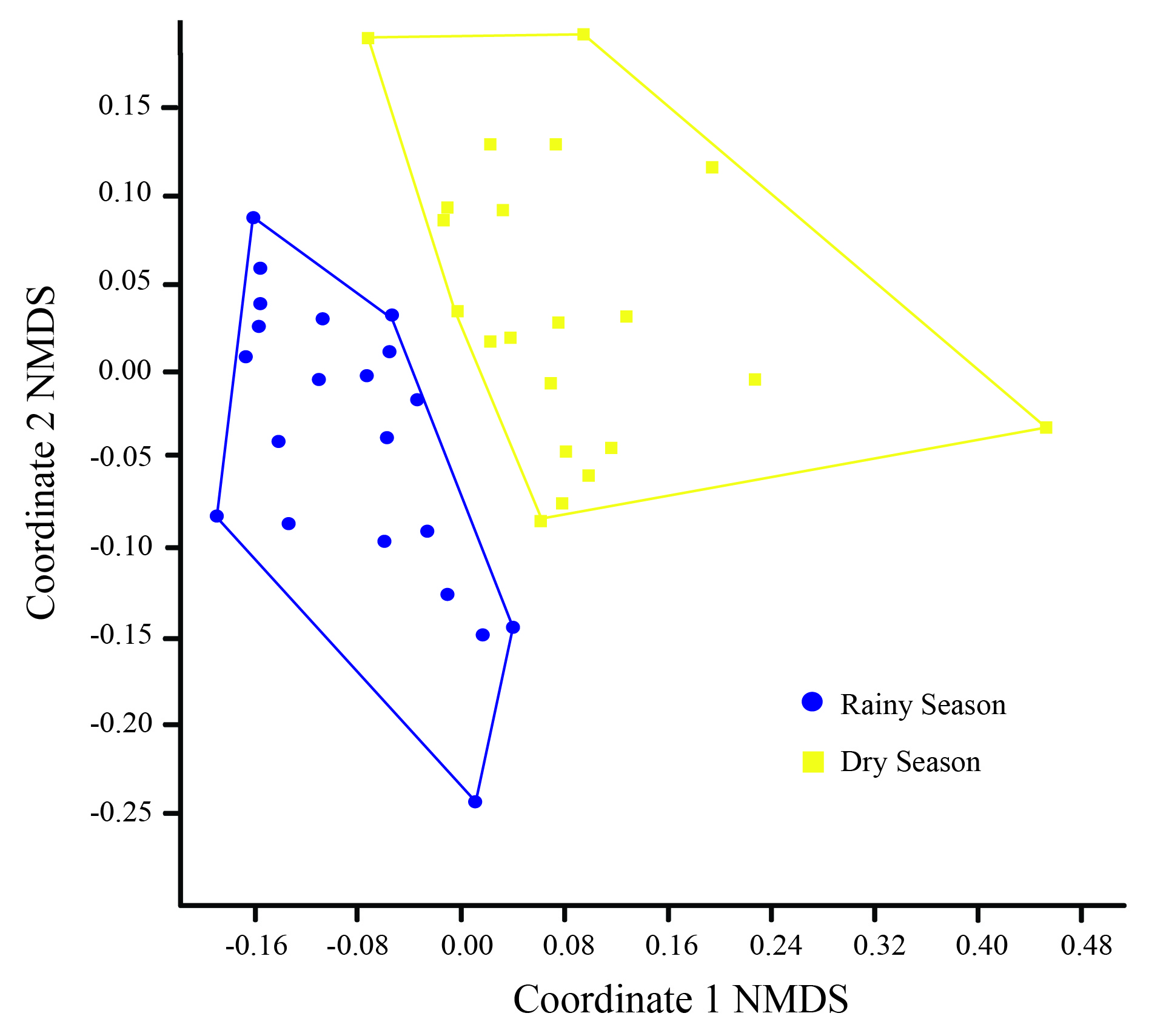


**Fig A.** Non Metric Multidimensional Scaling analysis (NMDS). Dung beetles collected in rainy season (December 2013 and March 2014) and in dry season (September 2013 and June 2014) at Serra do Cipó, State of Minas Gerais, Brazil.


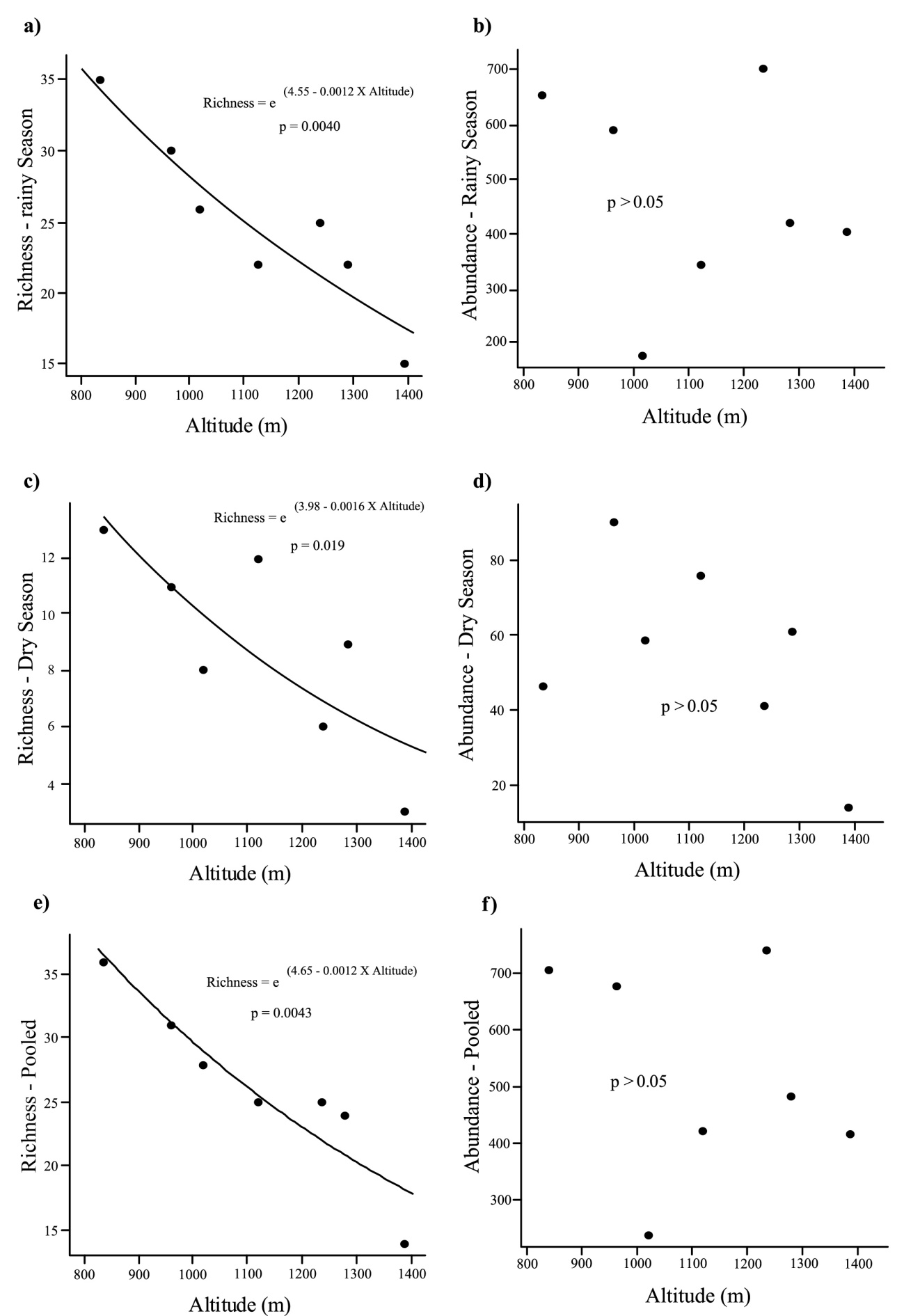


**Fig B.** Dung beetles richness and abundance against altitude at Serra do Cipó, State of Minas Gerais, Brazil. During the 2013-2014 rainy seasons (a and b), dry seasons (c and d), and pooled data presented in the original paper (e and f).


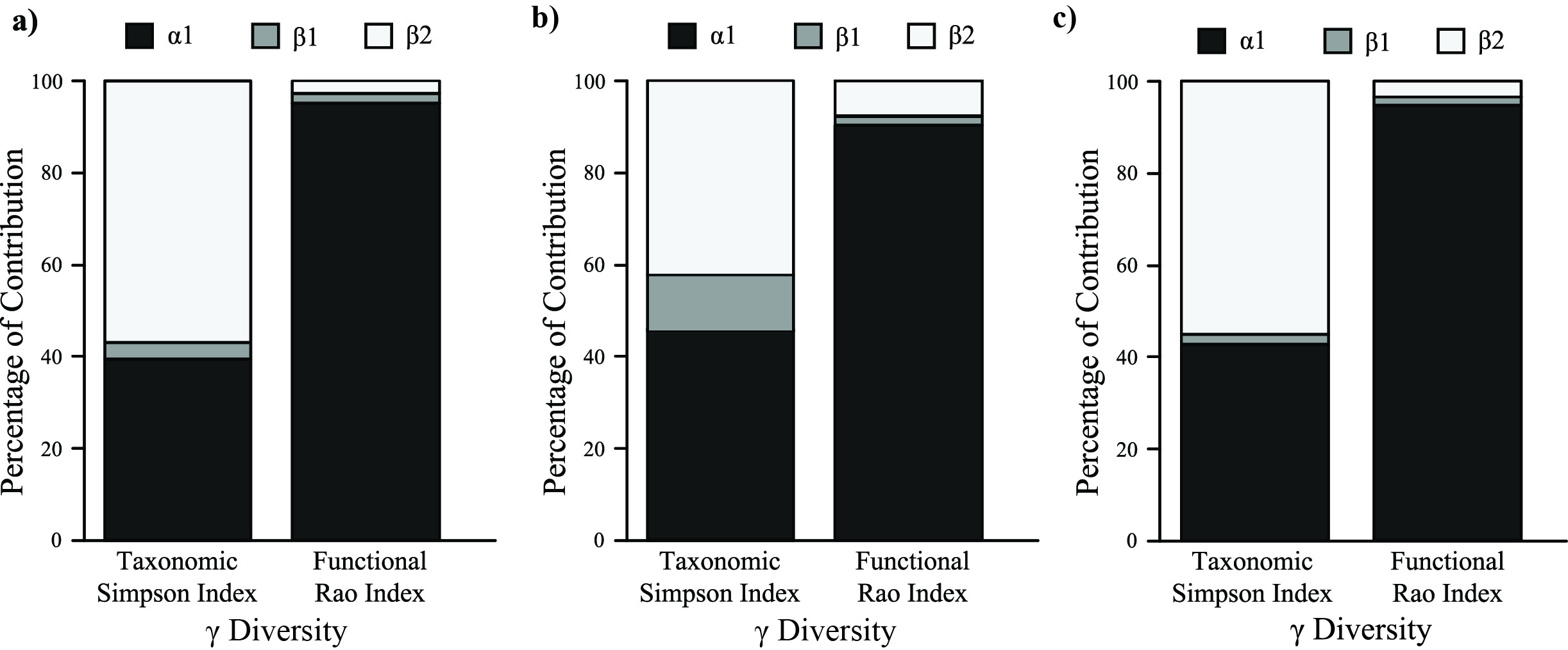


**Fig C.** Percentage of contribution of α1 (diversity of transects), β1 (diversity among transects) and β2 (diversity among altitudes) to γ taxonomic and functional diversities. a) rainy season; b) dry season and c) pooled data of an entire year (original paper).


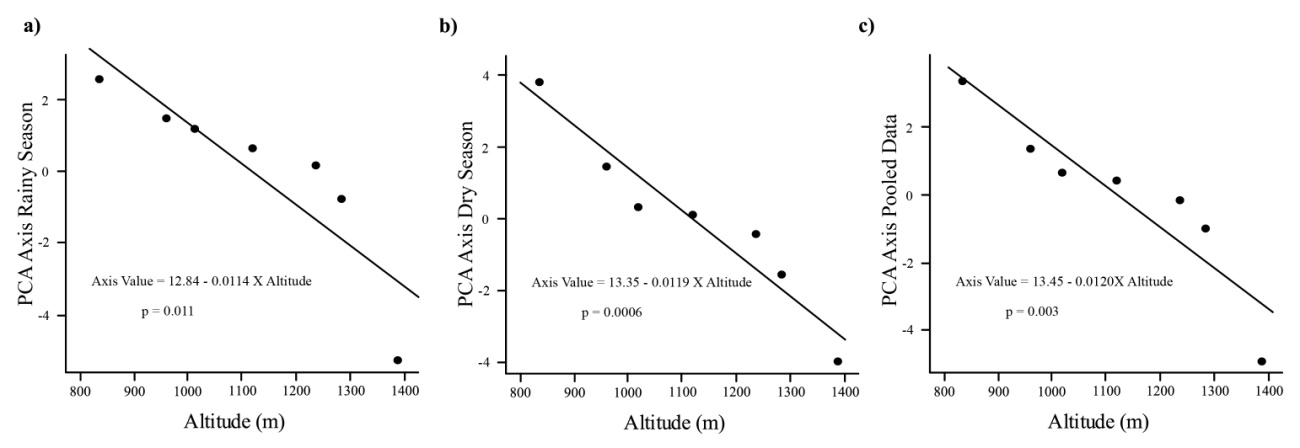


**Fig D.** Climatic axis resultant of Principal Components Analysis (PCA) against altitude. a) presents data of rainy season; b) presents data of dry season and c) presents data of the entire year (that was used in the paper statistical analysis, Appendix S1).

Table A. Results of additive partition of dung beetles taxonomic diversity separated by rainy/dry season and pooled (as in the paper). The expected diversities in null models were calculated 999 times by individual based randomization of the community data matrix and then compared with the observed values of diversities. Div. = Diversities; Obs. = Observed values; SES = Standardized effect sizes; Mean Sim. = Mean values of the 999 simulations; P = P values.

|  | **Div.** | **Obs.** | **SES** | **Mean Sim.** | **P** |
| --- | --- | --- | --- | --- | --- |
| **Rainy**  **Season** | **α 1** | 16.80 | -27.88 | 25.456 | **0.001** |
|  | **β 1** | 8.19 | -6.39 | 11.50 | **0.001** |
|  | **α 2** | 25.00 | -21.23 | 36.95 | **0.001** |
|  | **β 2** | 31.00 | 21.23 | 19.04 | **0.001** |
|  | **γ** | 56.00 | 0.00 | 56.00 | 1.000 |
| **Dry**  **Season** | **α 1** | 5.00 | -8.85 | 6.37 | **0.001** |
|  | **β 1** | 3.85 | -3.93 | 5.03 | **0.001** |
|  | **α 2** | 8.85 | -7.83 | 11.40 | **0.001** |
|  | **β 2** | 19.14 | 7.83 | 16.59 | **0.001** |
|  | **γ** | 28.00 | 0.00 | 28.00 | 1.000 |
| **Pooled** | **α 1** | 17.52 | -29.97 | 26.83 | **0.001** |
|  | **β 1** | 8.90 | -5.91 | 12.11 | **0.001** |
|  | **α 2** | 26.42 | -21.65 | 38.94 | **0.001** |
|  | **β 2** | 29.57 | 21.65 | 17.05 | **0.001** |
|  | **γ** | 56.00 | 0.00 | 56.00 | 1.000 |
